# Cytokine and Vasopressor Trajectories During Continuous Renal Replacement Therapy with oXiris, CytoSorb, or Standard Haemofilters in Sepsis-Associated Acute Kidney Injury: An Exploratory Retrospective Cohort Study

**DOI:** 10.3390/biomedicines14102230

**Published:** 2026-10-02

**Authors:** Tamara Mirela Porosnicu, Ciprian Gindac, Ahmed Elagez, Marius Papurica, Alexandru Silvius Pescariu, Felix Bratosin, Elena Hogea, Dorel Sandesc, Ovidiu Bedreag

**Affiliations:** 1 Department of Anaesthesia and Intensive Care, “Victor Babeș” University of Medicine and Pharmacy, Eftimie Murgu Square 2, 300041 Timișoara, Romania; mirela.porosnicu@umft.ro (T.M.P.); ciprian.gindac@umft.ro (C.G.); papurica.marius@umft.ro (M.P.); sandesc.dorel@umft.ro (D.S.); bedreag.ovidiu@umft.ro (O.B.); 2 Mercyhealth Family Medicine Residency Program–Rockford, Mercyhealth Alpine, 7702 North Alpine Road, Loves Park, IL 61111, USA; aelagez@mhemail.org; 3 Department VI—Cardiology, “Victor Babeș” University of Medicine and Pharmacy, Eftimie Murgu Square 2, 300041 Timișoara, Romania; 4 Department of Infectious Diseases, “Victor Babeș” University of Medicine and Pharmacy, Eftimie Murgu Square 2, 300041 Timișoara, Romania; 5 Department XIV, Discipline of Microbiology, “Victor Babeș” University of Medicine and Pharmacy, Eftimie Murgu Square 2, 300041 Timișoara, Romania; hogea.elena@umft.ro

**Keywords:** sepsis, hemoadsorption, continuous renal replacement therapy, vasoconstrictor agents, interleukin-6

## Abstract

**Background/Objectives:** This exploratory study described interleukin-6 (IL-6) and vasopressor-dependency trajectories during continuous renal replacement therapy (CRRT) with oXiris, CytoSorb, or standard haemofilters in sepsis-associated acute kidney injury, and their lagged and prognostic associations. **Methods:** This retrospective single-centre cohort included 48 adults receiving standard CRRT (*n* = 22), oXiris (*n* = 21), or CytoSorb (*n* = 5; analysed descriptively), assessed at baseline and 12, 24, and 48 h; devices were clinician-selected, implying confounding by indication. The vasopressor dependency index (VDI) was vasoactive-inotropic score/mean arterial pressure. **Results:** Twenty-eight-day survival was 13.6%, 33.3%, and 40.0%, respectively (*p* = 0.240). The unadjusted 24 h VDI decline was largest with CytoSorb (*p* = 0.018), but this group had much higher baseline IL-6 and VDI, and the contrast was attenuated and non-significant after baseline adjustment. No lagged association was detectable between IL-6 and subsequent VDI (β = 0.04, 95% confidence interval (CI) −0.04 to 0.13) or VDI and subsequent IL-6 (β = −0.05, 95% CI −0.20 to 0.09). In a 24 h landmark analysis, favourable haemodynamic status was associated with survival (odds ratio 7.57, 95% CI 1.75–32.69) without improving optimism-corrected discrimination beyond baseline severity and age (area under the curve 0.882). **Conclusions:** These hypothesis-generating findings do not establish a benefit of any device; early haemodynamic response criteria warrant prospective evaluation.

## 1. Introduction

Sepsis remains one of the leading causes of death in intensive care units worldwide, with an estimated 48.9 million incident cases and 11 million sepsis-related deaths in 2017, and its current definition emphasises life-threatening organ dysfunction caused by a dysregulated host response to infection [1,2]. Acute kidney injury develops in up to half of septic patients and, when it requires renal replacement therapy, is associated with mortality that frequently exceeds 50% [3]. Continuous renal replacement therapy (CRRT) is used for renal support in critically ill patients with sepsis-associated acute kidney injury. The 2021 Surviving Sepsis Campaign guidelines suggest either continuous or intermittent renal replacement therapy when indicated and identify insufficient evidence to recommend other blood purification techniques beyond their recommendation against polymyxin B haemoperfusion [4].

Over the past decade, extracorporeal blood purification has evolved from high-volume haemofiltration towards adsorptive strategies that target circulating cytokines, endotoxin, and other mediators through membrane surface treatment or dedicated sorbent cartridges [5]. The oXiris membrane combines an AN69 backbone with a polyethyleneimine coating and heparin grafting, and in a randomised crossover study reduced endotoxin and cytokine concentrations relative to a standard filter [6]. The CytoSorb polymer cartridge removed interleukin-6 (IL-6) across the device in a randomised trial of septic patients, but did not significantly lower systemic IL-6 concentrations or improve the assessed clinical outcomes [7], and its use during extracorporeal membrane oxygenation in severe COVID-19 did not improve survival [8]. A meta-analysis of randomised trials found no mortality benefit and raised a signal of harm, underlining the gap between biochemical efficacy and patient-centred outcomes [9]. Subsequent systematic reviews of oXiris have reported possible associations with lower mortality and reduced vasopressor requirements, but they rely largely on observational studies and rate the certainty of evidence as low or very low [10,11], and recent syntheses of CytoSorb and other haemoadsorption devices remain inconclusive regarding mortality benefit [12,13]. Membrane choice also blurs the boundary between “adsorptive” and “conventional” CRRT: polymethylmethacrylate (PMMA) haemofilters adsorb IL-6 and other cytokines in vitro and have been used as cytokine modulators in septic shock [14,15,16], so a comparator group that includes PMMA filters is not strictly non-adsorptive.

Most observational studies and small trials have reported physiological surrogates—falling cytokine concentrations, rising mean arterial pressure, and decreasing norepinephrine requirements—as evidence of effect, yet these signals are seldom analysed together as a coupled system. Similar dissociations between physiological signals and mortality have been described in cohorts of septic shock managed with haemofiltration during extracorporeal membrane oxygenation [17,18]. Such findings leave open a fundamental question: are changes in circulating cytokines associated with subsequent changes in vasopressor requirements, or do both simply mirror the resolution of the underlying septic process?

Addressing this question requires a longitudinal design in which biomarkers, vasopressor exposure, and organ dysfunction are measured repeatedly. Cross-lagged panel models can describe lagged associations between such variables, although they do not by themselves establish within-patient or causal effects [19]; ordinal desirability-of-outcome ranking (DOOR) endpoints retain information from every patient when binary mortality is insensitive in small samples [20]; and Firth-type penalisation stabilises logistic regression when events are few [21]. These methods are described in Section 2.4.

The present exploratory study applied this analytical framework to a real-world cohort of patients with sepsis-associated acute kidney injury treated with CRRT using standard haemofilters, the oXiris membrane or the CytoSorb cartridge, with serial measurements at initiation and after 12, 24, and 48 h. Its novelty lies in three elements that have not, to our knowledge, been combined previously in this setting: quantification of vasopressor exposure through a vasopressor dependency index that simultaneously accounts for norepinephrine, vasopressin, and the achieved arterial pressure; an exploratory examination of lagged associations between IL-6 kinetics, vasopressor dependency, lactate, and organ dysfunction; and a descriptive device comparison based on DOOR win probabilities and win ratios rather than on mortality alone.

Our objectives were therefore (i) to describe haemodynamic, inflammatory, and organ-dysfunction trajectories over the first 48 h of CRRT according to device strategy, acknowledging non-random allocation; (ii) to explore whether changes in IL-6 were associated with subsequent changes in vasopressor dependency, or vice versa, at the sampled intervals; (iii) to identify baseline and early-response variables associated with 28-day survival; and (iv) to evaluate whether early haemodynamic response or cytokine kinetics add prognostic value beyond baseline severity in penalised prediction models validated by bootstrap resampling and decision-curve analysis.

## 2. Materials and Methods

### 2.1. Study Design and Population

We conducted a retrospective, single-centre cohort study of consecutive adult patients treated with CRRT for sepsis-associated acute kidney injury in the intensive care unit of a tertiary university hospital in Timișoara, Romania, between 1 January 2021 and 31 December 2024. The study is reported in accordance with the Strengthening the Reporting of Observational Studies in Epidemiology (STROBE) recommendations [22]. Sepsis and septic shock were defined according to the Third International Consensus Definitions, and the decision to initiate CRRT and to select a particular haemofilter or adsorption cartridge was made by the attending intensivist according to local practice and device availability, without a formal allocation algorithm [1]. Acute kidney injury was defined and staged according to the Kidney Disease: Improving Global Outcomes (KDIGO) criteria [23]. CRRT was initiated for KDIGO stage 2 or 3 acute kidney injury in the presence of at least one of oligo-anuria with fluid overload, progressive azotaemia, or refractory metabolic acidosis or hyperkalaemia; the primary indication and the interval between intensive care unit admission and CRRT initiation were recorded for every patient. Supportive care followed the Surviving Sepsis Campaign guidelines with respect to antimicrobial therapy, fluid resuscitation, vasopressor titration to a mean arterial pressure of at least 65 mmHg, lung-protective ventilation, and glycaemic control [4].

Three treatment strategies were compared. The conventional group received CRRT with polyarylethersulfone (PAES) haemofilters (Prismaflex HF1400 set; Baxter, Deerfield, IL, USA; *n* = 12), polysulfone haemofilters on an Infomed HF440 platform (Infomed, Geneva, Switzerland; *n* = 5), or PMMA haemofilters (Hemofeel CH-1.8W; Toray, Tokyo, Japan; *n* = 5) on the Infomed platform. The membrane used was identified individually for every conventional-group patient. Because PMMA membranes have cytokine-adsorptive properties [14,15,16], all principal analyses were repeated after excluding the five PMMA-treated patients (Section 2.4). The oXiris group received CRRT with the oXiris set (Baxter), an AN69-based membrane with surface polyethyleneimine treatment and heparin grafting. The CytoSorb group received CRRT with a CytoSorb cartridge (CytoSorbents, Princeton, NJ, USA) installed in series before a PAES haemofilter on the Prismaflex platform. All circuits were run in continuous veno-venous haemodiafiltration or haemofiltration mode at a prescribed effluent dose of 25–37 mL/kg/h with a filtration fraction of 19–25%; anticoagulation was systemic unfractionated heparin in most patients, regional citrate in two patients, and none in four patients. Circuit duration was recorded for each patient. oXiris sets were used for up to 72 h and exchanged earlier only for circuit clotting; CytoSorb cartridges were exchanged every 24 h (median two cartridges per patient, range one to three); conventional haemofilters were exchanged for clotting or after 72 h. No patient changed device strategy during the 48 h observation window. The study was conducted in accordance with the Declaration of Helsinki and approved by the Local Commission of Ethics of the “Pius Brinzeu” Clinical Emergency Hospital, Timișoara, Romania (approval number I204; approval date 27 February 2025). The requirement for individual informed consent was waived by the ethics committee because of the retrospective design and full anonymisation of the dataset.

### 2.2. Eligibility Criteria and Group Allocation

Patients were eligible if they were aged 18 years or older, fulfilled Sepsis-3 criteria for sepsis with acute kidney injury requiring CRRT, and had haemodynamic, inflammatory, and organ-dysfunction data recorded at CRRT initiation and at 12 h thereafter. Patients were excluded if CRRT was started for a non-septic indication, if the circuit ran for less than 12 h, or if baseline procalcitonin or C-reactive protein had not been measured. Baseline procalcitonin and C-reactive protein were required because they characterise the inflammatory state at CRRT initiation, were used to describe the channelling of adsorptive devices towards hyperinflamed patients (confounding by indication), and were needed to derive the pre-specified secondary inflammatory outcomes (relative 24 h change); both analytes are part of the routine admission panel at our centre, so this criterion mainly excluded patients transferred from other hospitals in whom CRRT had been started before the first local laboratory panel. Baseline IL-6 was not an eligibility criterion. The requirement for at least 12 h of CRRT ensured that every analysed patient contributed at least one post-initiation assessment; patients who died or in whom CRRT was discontinued before the 12 h assessment were therefore not analysed and are enumerated in the flow diagram (Section 3.1). Patients in whom haemofiltration was delivered within an extracorporeal membrane oxygenation (ECMO) circuit for septic shock were not included; these patients have been reported separately in two earlier ECMO cohorts from our centre [17,18], and no participant in the present study was included in those publications. Patients in whom therapy was discontinued or who died before the 24 h or 48 h assessment were retained in the analysis with their available time points, and the reason for missing values was documented; one patient in the conventional group died before the 24 h assessment and lacked a baseline IL-6 measurement, so that 47 patients contributed 24 h data and baseline IL-6 values. Of 148 adult patients treated with CRRT in the unit during the study period, 100 were excluded: 62 because CRRT was started for a non-septic indication, 17 because haemofiltration was delivered within an ECMO circuit, 12 because the circuit ran for less than 12 h (death before the 12 h assessment in 8 and discontinuation of CRRT in 4), and 9 because baseline procalcitonin or C-reactive protein had not been measured (Section 3.1). The remaining 48 patients met all criteria and constituted the analysis population: 22 in the conventional group, 21 in the oXiris group, and 5 in the CytoSorb group. For pre-specified secondary analyses, the two adsorptive strategies were pooled (*n* = 26) and compared with conventional CRRT (*n* = 22). The numbers of patients contributing to each longitudinal analysis at each time point are reported in Section 3.1 and in the figure and table captions.

Group allocation reflected clinical practice during the study period rather than randomisation. Adsorptive devices were preferentially used in patients with septic shock and marked hyperinflammation, as reflected by higher baseline procalcitonin in the CytoSorb group, whereas conventional filters were more frequently used in patients with predominant azotaemia. Because such channelling introduces confounding by indication, all comparative analyses were interpreted as associations, effect estimates were adjusted for baseline severity where model size allowed, and a proportional-odds model of the DOOR outcome was fitted with baseline SOFA score as a covariate. Subgroup analyses were pre-specified according to baseline IL-6 (above or below the median of vasopressor-dependent patients), baseline SOFA score (≥12 versus <12), age (≥65 versus <65 years), baseline norepinephrine dose (≥0.3 versus <0.3 µg/kg/min), baseline lactate (≥2 versus <2 mmol/L), acute respiratory distress syndrome, SARS-CoV-2 infection, and obesity.

### 2.3. Variables, Outcomes, and Data Collection

Demographic data, comorbidities, infection source, Acute Physiology and Chronic Health Evaluation (APACHE) II score, and Sequential Organ Failure Assessment (SOFA) score were extracted from the electronic medical record; the SOFA score was recorded at each of the four time points and APACHE II at admission [24,25]. At 0, 12, 24, and 48 h after CRRT initiation, we recorded mean arterial pressure (MAP), heart rate, norepinephrine dose (µg/kg/min), vasopressin dose, urine output, temperature, arterial pH, bicarbonate and lactate, and the plasma concentrations of IL-6, procalcitonin, C-reactive protein, ferritin, white blood cells, creatinine, and urea. IL-6, procalcitonin, and ferritin were measured in the hospital central laboratory as part of routine care by electrochemiluminescence immunoassay (Elecsys IL-6, Elecsys BRAHMS PCT and Elecsys Ferritin on a cobas e 601 analyser; Roche Diagnostics, Mannheim, Germany), with measuring ranges of 1.5–5000 pg/mL, 0.02–100 ng/mL and up to 4787 ng/mL (laboratory reporting limit without dilution), respectively; C-reactive protein was measured by immunoturbidimetry (cobas c 501, Roche). Samples were not diluted beyond the analyser protocol, so values at the upper limit represent assay ceilings rather than true concentrations. Vasopressor exposure was summarised as the vasoactive-inotropic score (VIS = 100 × norepinephrine [µg/kg/min] + 10,000 × vasopressin [U/kg/min]), a composite of the vasoactive drugs recorded in this cohort, using weights from the vasoactive-inotropic score evaluated in adult cardiac surgery [26]. Because vasopressor dose is only interpretable relative to the pressure it achieves, we defined the vasopressor dependency index (VDI) as VIS divided by MAP (mmHg); a decrease in VDI therefore indicates less vasoactive support for the same or a higher arterial pressure. Because VDI incorporates MAP in its denominator and the VIS weights were derived in cardiac surgery, vasopressor exposure was additionally expressed as the norepinephrine-equivalent dose (norepinephrine in µg/kg/min plus vasopressin in U/min multiplied by 2.5) [27], and the norepinephrine-equivalent dose and MAP were analysed separately from VDI in sensitivity analyses.

The primary clinical outcome was survival at 28 days after CRRT initiation. The primary physiological outcome was the absolute change in VDI between 0 and 24 h among patients who were vasopressor-dependent at baseline (*n* = 36); a haemodynamic response was defined as a VDI reduction of at least 50% at 24 h. The 50% threshold was chosen a priori as a clinically recognisable halving of vasoactive support and has not been externally validated; a sensitivity definition based on a reduction in the norepinephrine-equivalent dose of at least 50% was therefore also analysed. Because 12 patients received no vasopressor at CRRT initiation, a cohort-wide dichotomous variable termed favourable haemodynamic status at 24 h was also defined as a VDI reduction of at least 50% in vasopressor-dependent patients or persistence of a vasopressor-free state in patients without vasopressors at baseline. Because this composite combines patients who improved with patients who simply remained vasopressor-free, its two components are reported separately, and the response criterion restricted to vasopressor-dependent patients is used in sensitivity analyses. Secondary outcomes were vasopressor-free status at 48 h, the change in MAP and lactate at 24 h, the relative change in IL-6, procalcitonin, C-reactive protein and ferritin at 24 h, an IL-6 reduction of at least 50% at 24 h, and the change in SOFA score at 24 and 48 h. The DOOR outcome was an ordinal four-level ranking: (1) alive at day 28, vasopressor-free at 48 h, and SOFA improved by at least 2 points; (2) alive and vasopressor-free at 48 h without a 2-point SOFA improvement; (3) alive but vasopressor-dependent at 48 h or with unknown 48 h status; and (4) dead by day 28 [20]. Data were entered in a structured spreadsheet and screened for implausible values: one norepinephrine value of 1025 µg/kg/min, one pH value of 1.31 and two lactate values above 250 mmol/L at 48 h were regarded as transcription errors and set to missing; vasopressin doses recorded above 0.1 U/min were interpreted as units per hour and converted after verification against the infusion-pump records and intensive care unit flow sheets of the six patients concerned, all of which documented infusions of 1.8–2.4 U/h; the four implausible values could not be verified against source records and remained missing. IL-6 (5000 pg/mL), procalcitonin (100 ng/mL) and ferritin (4787 ng/mL) values at the upper limit of the assay were retained at the ceiling value and flagged; IL-6 reached the ceiling in 5 of 178 measurements (three patients), procalcitonin in 11 of 186 (seven patients), and ferritin in 6 of 175 (five patients). Relative changes involving a ceiling value are therefore bounded, and analyses of IL-6 change were repeated after excluding patients with any ceiling value.

### 2.4. Statistical Analysis

Continuous variables are reported as median with interquartile range and categorical variables as counts and percentages, with values in tables rounded to one decimal place unless a finer resolution was statistically meaningful. Distributions were inspected with quantile–quantile plots and the Shapiro–Wilk test; because most variables were skewed, between-group comparisons used the Kruskal–Wallis test for three groups and the Mann–Whitney U test for two groups, with effect sizes expressed as Hodges–Lehmann median differences with distribution-free 95% confidence intervals (CIs). Categorical variables were compared with Fisher’s exact test, extended by Monte-Carlo permutation (20,000 replicates) for tables larger than 2 × 2. Longitudinal trajectories of log-transformed VDI (after adding 0.05 to accommodate zero values), MAP, log IL-6, log procalcitonin, C-reactive protein, log ferritin, lactate, bicarbonate, and SOFA were analysed with linear mixed-effects models including a random intercept per patient, time (per 24 h) as a continuous fixed effect, group, and the group × time interaction; group-specific slopes with 95% CIs were derived from the fixed-effect covariance matrix, and the interaction was tested by likelihood-ratio test of nested models fitted by maximum likelihood. Because the observed trajectories were not strictly linear, the linear slope is reported as an average rate of change over the 48 h window, that is, as a summary of the overall direction and magnitude of each group’s trajectory rather than as a description of its shape. In a sensitivity analysis, time was therefore entered as a categorical factor (0, 12, 24, and 48 h) with the same random-intercept structure and a group × time interaction, which imposes no functional form on the trajectory; the interaction was again tested by likelihood-ratio test, and the categorical-time contrasts of the change from baseline to 24 h and to 48 h between each adsorptive strategy and conventional CRRT are reported alongside the linear slope contrasts (Section 3.3). Subgroup heterogeneity was tested with interaction terms in linear models fitted on rank-transformed outcomes. Because the groups differed at baseline, follow-up values of VDI, norepinephrine-equivalent dose, IL-6, MAP, and SOFA were additionally compared between groups after adjustment for the corresponding baseline value by analysis of covariance, on the log scale for VDI, norepinephrine-equivalent dose, and IL-6 (reported as ratios of geometric means) and on the raw scale for MAP and SOFA (adjusted mean differences), with 95% CIs. Because VDI and the norepinephrine-equivalent dose were log-transformed after adding offsets of 0.05 and 0.01, respectively, the exponentiated estimates for these two variables are ratios of geometric means of the offset-transformed quantities (VDI + 0.05 and norepinephrine-equivalent dose + 0.01) and not of the unmodified variables; they are labelled as such in the tables and should be interpreted accordingly, whereas the IL-6 ratios are unaffected because no offset was applied. Between-group *p*-values for the primary and secondary outcomes (Section 3.2) were additionally adjusted for multiplicity with the Benjamini–Hochberg procedure [28].

Associations between changes in continuous variables were assessed using Spearman rank correlations with available pairs. Time to the first VDI reduction of at least 50% among patients receiving vasopressors at baseline was described with discrete-time cumulative incidence functions (Aalen–Johansen estimator) at 12, 24, and 48 h, in which death before response was treated as a competing event rather than as censoring, because death precludes a subsequent response [29]; cumulative incidence curves were compared with Gray’s test. These cumulative estimates describe first response during follow-up and differ from response status at the 24 h assessment.

Lagged associations were explored with a first-order cross-lagged panel model, which is presented as a description of lagged associations rather than as a test of temporal precedence. Log IL-6, log VDI, SOFA, and lactate were standardised across all observations, and each variable at time t + 1 was regressed on all four variables at time t together with indicator variables for the transition interval (0–12, 12–24, and 24–48 h), pooling 129 transitions from 47 patients; standard errors were clustered by patient, and a sensitivity model additionally adjusted for adsorptive therapy [19]. Because 12 h and 24 h intervals were pooled, the model was also refitted separately for the two 12 h transitions (0–12 and 12–24 h) and for the single 24 h transition (24–48 h). The difference between reciprocal cross-lagged coefficients was estimated with a patient-level cluster bootstrap (1000 replicates). Device strategies were compared on the DOOR outcome with the win probability (the probability that a randomly selected patient in one group has a better rank than a randomly selected patient in the comparator, ties counted as one half), the win ratio, and Mann–Whitney tests, with bootstrap 95% CIs (2000 replicates) [20,30]; an adjusted proportional-odds model included baseline SOFA. Associations with 28-day survival were quantified as odds ratios (ORs) per one standard deviation from Firth-penalised logistic regression, which yields finite estimates when events are few, with penalised likelihood-ratio *p*-values in which the restricted model fixes the coefficient at zero while retaining the full information matrix in the Jeffreys penalty [21]. All variables measured at 24 or 48 h were treated as landmark variables conditional on survival with available data at that time point, not as baseline predictors [31]. Nested 24 h landmark prediction models were built sequentially (M1, baseline SOFA and age; M2, M1 plus favourable haemodynamic status at 24 h; M3, M2 plus the 24 h IL-6 log-ratio and adsorptive device) and evaluated by apparent and optimism-corrected area under the receiver operating characteristic curve (AUC; 500 bootstrap resamples with refitting), calibration slope, Brier score and Nagelkerke R^2^; clinical utility was assessed with decision-curve analysis across threshold probabilities of 5–70% [32]. With 12 events, M3 (five predictors) falls below the recommended events-per-variable ratio and is reported only in the Supplementary Materials (Section 3.9). Restricted cubic splines with three knots at the 10th, 50th, and 90th percentiles were used as an additional check of non-linearity for baseline SOFA and log IL-6 in Firth models. Missing data were sparse before 48 h (≤3 values per variable) and were handled by available-case analysis; mixed models used all available observations under the missing-at-random assumption, and the ten patients without a usable 48 h assessment (six with no 48 h record and four with incomplete records; nine of the ten died) were incorporated into the DOOR outcome by rank rather than excluded. Because missingness at 48 h was strongly associated with death, sensitivity analyses repeated the mixed models in 48 h completers (*n* = 38) and re-estimated 48 h vasopressor-free status under a worst-case assumption in which all patients without a usable 48 h assessment were classified as vasopressor-dependent. Sensitivity analyses therefore comprised (i) exclusion of PMMA-treated patients, (ii) baseline-adjusted follow-up values, (iii) norepinephrine-equivalent dose and MAP in place of VDI, (iv) 48 h completers and worst-case imputation, (v) exclusion of patients with assay-ceiling values, (vi) interval-specific cross-lagged models, and (vii) mixed models with time entered as a categorical factor. Prediction-model reporting followed the TRIPOD statement [33]. All tests were two-sided with a significance threshold of 0.05. Benjamini–Hochberg-adjusted *p*-values are reported for the primary and secondary outcomes (Section 3.2); all other secondary, subgroup, and device-specific analyses are unadjusted, and because of the small sample—particularly the five-patient CytoSorb group—they are regarded as descriptive and hypothesis-generating. Analyses were performed in Python 3.11 (Python Software Foundation, Wilmington, DE, USA) with pandas 3.0, SciPy 1.17, statsmodels 0.15, and scikit-learn 1.8.

## 3. Results

### 3.1. Cohort Characteristics

The flow of patients through the study, including the number of potentially eligible CRRT patients, the exclusions and the reasons for exclusion, is shown in Figure 1. Baseline characteristics are presented in Table 1. The cohort included 48 patients: 22 receiving conventional CRRT, 21 oXiris, and 5 CytoSorb. Median age was 65.5 years, 58.3% were male, 79.2% had septic shock, and 75.0% received vasopressors at baseline. Median APACHE II and SOFA scores were 23.0 and 12.0, respectively. Baseline procalcitonin differed between groups (*p* = 0.030), with the highest median in the CytoSorb group, whereas urea was highest with conventional CRRT (*p* = 0.018). Baseline VDI and prescribed effluent dose did not differ significantly (*p* = 0.063 and *p* = 0.317), although the CytoSorb group was younger and had higher norepinephrine, VDI, IL-6, and procalcitonin values, and lower urea, a pattern that reflects confounding by indication. Of the 22 conventional-group patients, 12 received PAES, 5 polysulfone, and 5 PMMA haemofilters. Acute kidney injury was KDIGO stage 3 in 40 patients (83.3%) and stage 2 in 8 (16.7%); the median interval from intensive care unit admission to CRRT initiation was 2.0 (1.0–3.2) days, and oligo-anuria with fluid overload was the most frequent primary indication (28 patients, 58.3%), followed by progressive azotaemia (11, 22.9%) and refractory acidosis or hyperkalaemia (9, 18.8%). Pneumonia was the source of infection in 29 patients (60.4%), intra-abdominal infection in 10 (20.8%), urinary tract infection in 4 (8.3%), and skin, soft-tissue, or other sources in 5 (10.4%), without significant differences between groups. The numbers of patients contributing to the longitudinal analyses at 0, 12, 24, and 48 h were 48, 48, 47, and 38 for VDI, MAP, and SOFA (36 for lactate at 48 h) and 47, 47, 47, and 37 for IL-6; the ten patients without a usable 48 h assessment comprised eight conventional and two oXiris patients, of whom nine died before or at the 48 h assessment.

### 3.2. Primary and Secondary Outcomes

Clinical and physiological outcomes are presented in Table 2. Twenty-eight-day survival was 13.6% with conventional CRRT, 33.3% with oXiris, and 40.0% with CytoSorb (*p* = 0.240); pooled adsorptive survival was 34.6% versus 13.6% (*p* = 0.180). In unadjusted analysis the 24 h VDI change differed between groups (*p* = 0.018), with a CytoSorb-versus-conventional Hodges–Lehmann difference of −0.46 (95% CI −0.84 to −0.12; *p* = 0.011) and an oXiris-versus-conventional difference of −0.08 (95% CI −0.31 to 0.12; *p* = 0.308); the change in norepinephrine-equivalent dose showed the same pattern (*p* = 0.021), whereas the change in MAP did not differ (*p* = 0.412). The CytoSorb contrast should be read against this group’s much higher baseline VDI (Table 1) and is attenuated after baseline adjustment (Section 3.4). Lactate change differed between groups (*p* = 0.035; pooled difference −0.60 mmol/L, 95% CI −1.32 to −0.07). Nine of 35 patients met the 24 h haemodynamic response criterion. IL-6 changes did not differ significantly (*p* = 0.499); the 48 h SOFA change did (*p* = 0.047). After Benjamini–Hochberg adjustment across the eleven outcomes in Table 2, no between-group difference remained significant (all adjusted *p* ≥ 0.116).

### 3.3. Longitudinal Trajectories over 48 H

Group-specific mixed-model slopes are presented in Table 3. The log-transformed VDI slope was −0.44 per 24 h with CytoSorb, −0.02 with oXiris and +0.03 with conventional CRRT (interaction *p* = 0.050; CytoSorb-versus-conventional contrast *p* = 0.015); the norepinephrine-equivalent dose showed a similar but non-significant pattern (interaction *p* = 0.078). Group-by-time interactions were significant for log IL-6 (*p* = 0.034) and SOFA (*p* = 0.012). MAP increased over time (*p* = 0.040), without a significant interaction (*p* = 0.984). Procalcitonin and C-reactive protein decreased over time (both *p* < 0.001); lactate, ferritin, and bicarbonate showed no significant group-by-time interaction. These slopes describe unadjusted within-group trajectories from different starting values; baseline-adjusted comparisons of the 24 h and 48 h values are reported in Section 3.4. Because the median trajectories were visibly non-linear (Figure 2), the linear slopes should be read as average rates of change over 48 h rather than as descriptions of trajectory shape. When time was entered as a categorical factor, the group × time interaction *p*-values were 0.074 for log VDI, 0.096 for the log norepinephrine-equivalent dose, 0.041 for log IL-6, 0.017 for SOFA, and 0.93 for MAP, and the CytoSorb-versus-conventional contrasts for the change in log(VDI + 0.05) were −0.59 (95% CI −1.20 to 0.02; *p* = 0.058) from 0 to 24 h and −1.04 (95% CI −1.83 to −0.25; *p* = 0.010) from 0 to 48 h, with the oXiris and pooled adsorptive contrasts close to the null at both time points (Table 4); the categorical-time model therefore led to the same conclusions as the linear model.

Median VDI and IL-6 trajectories are shown in Figure 2. In the CytoSorb group, median VDI decreased from 1.17 at baseline to 0.64, 0.74, and 0.28 at 12, 24, and 48 h, respectively; median IL-6 decreased from 1351 to 1000, 160, and 88 pg/mL. Corresponding baseline and 48 h VDI values were 0.26 and 0.20 with conventional CRRT, and 0.19 and 0.10 with oXiris. The numbers contributing measurements at each time point are provided in the caption. The much higher CytoSorb starting values mean that part of the subsequent decline is expected from regression to the mean alone.

Figure 3 shows the cumulative incidence of a first haemodynamic response with death before response treated as a competing event. Fifteen of 36 patients (41.7%) responded during follow-up, and five (13.9%) died before any response. At 24 h, the cumulative incidence of response was 31.3% with conventional CRRT, 40.0% with oXiris, and 60.0% with CytoSorb, and at 48 h, 31.3%, 46.7%, and 60.0%, respectively; the cumulative incidence of death before response at 48 h was 25.0%, 6.7% and 0%. Numbers at risk (alive without prior response) declined from 36 at baseline to 27 at 24 h and 17 at 48 h. Gray’s test was not significant for the three groups (*p* = 0.561) or for pooled adsorptive versus conventional therapy (*p* = 0.352). These competing-risk estimates are slightly lower than Kaplan–Meier estimates that treat death as censoring (32.7%, 40.0%, and 60.0% at 24 h).

### 3.4. Sensitivity and Subgroup Analyses

Sensitivity analyses of the principal device contrasts are summarised in Table 4. Excluding the five PMMA-treated patients from the conventional group (leaving 17 patients treated with PAES or polysulfone membranes, 11 of whom were vasopressor-dependent with 24 h data) left the unadjusted estimates essentially unchanged. After adjustment for the corresponding baseline value, however, the CytoSorb-versus-conventional contrasts were attenuated and no longer significant for VDI (ratio of geometric means of VDI + 0.05 at 24 h: 0.71, 95% CI 0.33 to 1.52; *p* = 0.37), norepinephrine-equivalent dose (ratio of geometric means of the dose + 0.01: 0.74, 95% CI 0.35 to 1.57; *p* = 0.43), IL-6 (0.62, 95% CI 0.28 to 1.38; *p* = 0.23) and SOFA at 48 h (adjusted difference −1.4 points, 95% CI −3.3 to 0.5; *p* = 0.14), and the oXiris and pooled adsorptive contrasts remained close to the null. Using the norepinephrine-equivalent dose instead of VDI, 8 of 35 vasopressor-dependent patients (3 conventional, 3 oXiris, and 2 CytoSorb; *p* = 0.79) met the 50% response criterion, and this definition was associated with 28-day survival in the same direction as the VDI definition (24 h landmark OR 9.61, 95% CI 1.46 to 63.2; *p* = 0.011). Restricting the mixed models to the 38 patients with a complete 48 h assessment gave group-by-time interaction *p*-values of 0.071 for VDI, 0.052 for IL-6, and 0.019 for SOFA, and under the worst-case assumption that all ten patients without a usable 48 h assessment remained vasopressor-dependent, vasopressor-free status at 48 h was 27.3%, 28.6%, and 20.0% in the three groups (*p* = 0.96). Excluding the three patients with an IL-6 value at the assay ceiling did not change the between-group comparison of IL-6 change (*p* = 0.47) or the lagged IL-6-to-VDI coefficient (β = 0.05, 95% CI −0.04 to 0.14). The categorical-time mixed models are also summarised in Table 4: the CytoSorb-versus-conventional contrast for the change in log(VDI + 0.05) was −0.59 (95% CI −1.20 to 0.02) at 24 h and −1.04 (95% CI −1.83 to −0.25) at 48 h, and the oXiris and pooled adsorptive contrasts were close to the null, in agreement with the linear-slope contrasts.

**Table 4:** Sensitivity analyses of the device contrasts for the 24 h change in vasopressor dependency and related outcomes.

Analysis	CytoSorb vs. Conventional CRRT	oXiris vs. Conventional CRRT	Pooled Adsorptive vs. Conventional CRRT
24 h VDI change, primary analysis (HL difference) ^1^	−0.46 (−0.84 to −0.12); *p* = 0.011	−0.08 (−0.31 to 0.12); *p* = 0.308	−0.14 (−0.44 to 0.02); *p* = 0.077
24 h VDI change, PMMA-treated patients excluded (HL difference) ^1^	−0.44 (−0.86 to −0.09); *p* = 0.016	−0.07 (−0.33 to 0.14); *p* = 0.36	−0.13 (−0.46 to 0.04); *p* = 0.10
VDI at 24 h adjusted for baseline VDI (ratio of geometric means of VDI + 0.05) ^1^	0.71 (0.33 to 1.52); *p* = 0.37	0.95 (0.63 to 1.43); *p* = 0.80	0.90 (0.61 to 1.33); *p* = 0.60
24 h change in norepinephrine-equivalent dose (HL difference, µg/kg/min) ^1^	−0.31 (−0.62 to −0.06); *p* = 0.019	−0.03 (−0.14 to 0.06); *p* = 0.42	−0.08 (−0.24 to 0.02); *p* = 0.11
Norepinephrine-equivalent dose at 24 h adjusted for baseline (ratio of geometric means of dose + 0.01) ^1^	0.74 (0.35 to 1.57); *p* = 0.43	0.97 (0.66 to 1.42); *p* = 0.87	0.92 (0.64 to 1.32); *p* = 0.65
24 h MAP change (HL difference, mmHg)	5 (−6 to 14); *p* = 0.41	2 (−5 to 9); *p* = 0.55	3 (−4 to 9); *p* = 0.44
MAP at 24 h adjusted for baseline MAP (difference, mmHg)	3 (−6 to 12); *p* = 0.49	1 (−5 to 7); *p* = 0.71	2 (−4 to 7); *p* = 0.57
IL-6 at 24 h adjusted for baseline IL-6 (ratio of geometric means)	0.62 (0.28 to 1.38); *p* = 0.23	1.21 (0.72 to 2.04); *p* = 0.47	1.06 (0.66 to 1.71); *p* = 0.80
SOFA at 48 h adjusted for baseline SOFA (difference, points)	−1.4 (−3.3 to 0.5); *p* = 0.14	0.6 (−0.7 to 1.9); *p* = 0.35	0.2 (−1.0 to 1.4); *p* = 0.72
Mixed-model VDI slope contrast per 24 h, 48 h completers only (*n* = 38)	−0.41 (−0.77 to −0.05); *p* = 0.026	−0.04 (−0.23 to 0.15); *p* = 0.68	−0.11 (−0.29 to 0.07); *p* = 0.22
Categorical-time mixed-model contrast, change in log(VDI + 0.05) from 0 to 24 h	−0.59 (−1.20 to 0.02); *p* = 0.058	−0.06 (−0.37 to 0.25); *p* = 0.70	−0.17 (−0.46 to 0.12); *p* = 0.25
Categorical-time mixed-model contrast, change in log(VDI + 0.05) from 0 to 48 h	−1.04 (−1.83 to −0.25); *p* = 0.010	−0.09 (−0.49 to 0.31); *p* = 0.66	−0.27 (−0.65 to 0.11); *p* = 0.16

Negative differences and ratios below 1 indicate lower values with the adsorptive strategy. HL, Hodges–Lehmann median difference with distribution-free 95% CI and Mann–Whitney *p*-value; baseline-adjusted estimates from analysis of covariance on the log scale (ratios of geometric means) or raw scale (mean differences) with 95% CIs. For VDI and the norepinephrine-equivalent dose, which were log-transformed after adding 0.05 and 0.01, respectively, the ratios are ratios of geometric means of the offset-transformed variables (VDI + 0.05 and norepinephrine-equivalent dose + 0.01), not of the unmodified variables. Categorical-time contrasts are differences in the change in log(VDI + 0.05) from baseline (fixed-effect estimates with 95% CIs) from random-intercept mixed models with time as a categorical factor (0, 12, 24, and 48 h) and a group × time interaction; the corresponding interaction *p*-value was 0.074. ^1^ Vasopressor-dependent patients with 24 h data (15, 15 and 5 per group; 11 conventional patients after exclusion of PMMA-treated patients). All analyses are exploratory and unadjusted for multiplicity; the CytoSorb group contains five patients. Abbreviations: CI, confidence interval; CRRT, continuous renal replacement therapy; IL-6, interleukin-6; MAP, mean arterial pressure; PMMA, polymethylmethacrylate; SOFA, Sequential Organ Failure Assessment; VDI, vasopressor dependency index.

Subgroup comparisons are presented in Supplementary Table S1. Among 35 vasopressor-dependent patients with 24 h data, the pooled adsorptive-versus-conventional VDI difference was −0.14 (95% CI −0.44 to 0.02; *p* = 0.077). The largest differences were observed with baseline norepinephrine ≥ 0.3 µg/kg/min (−0.41), lactate ≥ 2 mmol/L (−0.36), and IL-6 ≥ 114 pg/mL (−0.26); each confidence interval included zero. All interaction *p*-values were ≥0.264, and none of these strata should be regarded as identifying a subgroup that benefits. In additional strata, the difference was −0.18 among 32 patients without SARS-CoV-2 infection (95% CI −0.48 to −0.02; *p* = 0.030); interaction *p*-values were 0.804 for ARDS and 0.854 for obesity.

### 3.5. Associations with 28-Day Survival

Univariable associations with survival are presented in Table 5; all early-response variables are 24 h or 48 h landmark variables conditional on survival with available data at that time point, and the corresponding ORs must not be read as baseline predictors. Survivors had lower baseline SOFA scores (median 9.0 versus 12.0; *p* = 0.002) and urea (*p* = 0.012). The OR for survival per standard deviation of baseline SOFA was 0.27 (95% CI 0.11 to 0.65). Haemodynamic response at 24 h was present in 71.4% of survivors versus 14.3% of non-survivors among initially vasopressor-dependent patients (OR 11.98, 95% CI 1.85 to 77.43). Cohort-wide favourable haemodynamic status had an OR of 7.57 (95% CI 1.75 to 32.69); its two components contributed 5 survivors among the 9 vasopressor-dependent responders and 4 survivors among the 9 patients who remained vasopressor-free, compared with 2 survivors among the 26 vasopressor-dependent non-responders and 1 among the 3 patients who newly required vasopressors by 24 h. IL-6 reduction ≥ 50% was present in 33.3% and 28.6%, respectively (*p* = 0.731). The OR for adsorptive therapy was 3.02 (95% CI 0.73 to 12.45).

Survival by early-response status is shown in Figure 4 as an unadjusted, descriptive landmark analysis. Survival was 50.0% with favourable haemodynamic status at 24 h versus 10.3% without it (9/18 versus 3/29; *p* = 0.005), 28.6% with an IL-6 reduction ≥ 50% versus 24.2% without it (4/14 versus 8/33; *p* = 0.731), and 46.2% among patients free of vasopressors at 48 h versus 20.0% among those still receiving them (6/13 versus 5/25; *p* = 0.135).

In additional spline analyses, baseline SOFA was associated with survival after adjustment for age (linear term *p* < 0.001; non-linearity *p* = 0.853). Predicted survival was 85.5%, 23.3%, and 2.7% at SOFA scores of 5, 11, and 16, respectively. Baseline IL-6 was not significant after adjustment for SOFA and age (linear *p* = 0.176; non-linear *p* = 0.655).

### 3.6. Correlations Between Early Changes

Correlations between early changes are presented in Table 6. VDI change correlated with MAP change (ρ = −0.35; *p* = 0.040), and MAP change correlated with the 48 h SOFA change (ρ = −0.33; *p* = 0.041). The VDI–SOFA correlation was ρ = 0.36 (*p* = 0.060). Relative IL-6 change was not significantly correlated with VDI change (ρ = 0.28; *p* = 0.102) or SOFA change (ρ = 0.10; *p* = 0.549). Additional correlations of C-reactive protein change with procalcitonin and ferritin changes were ρ = 0.38 (*p* = 0.008) and ρ = 0.50 (*p* < 0.001), respectively.

The response map in Figure 5 includes 35 initially vasopressor-dependent patients. Phenotypes were classified with the same raw-scale criteria used in Table 2 and Table 5 (VDI and IL-6 reductions of at least 50%). Both axes display the raw 24 h ratio (value at 24 h divided by the baseline value) on a logarithmic scale without any offset, so that the dashed lines at a ratio of 0.5 correspond exactly to the 50% response definitions; all nine haemodynamic responders counted in Table 2 and Table 5, including one oXiris survivor with a low baseline VDI (0.10, falling to 0.04 at 24 h, a 60% reduction), therefore lie below the horizontal dashed line, and no exception is required. The phenotypes comprised 5 concordant responders, 7 cytokine-only responders, 4 haemodynamic-only responders, and 19 non-responders; survival was 60.0%, 0%, 50.0%, and 10.5%, respectively. Adsorptive devices accounted for 4/5, 4/7, 2/4, and 10/19 patients in these categories. The Spearman correlation between the logarithms of the IL-6 and VDI ratios was ρ = 0.34 (*p* = 0.049). Survival was 5/9 among patients in the haemodynamic-response quadrants and 2/26 in the other quadrants, consistent with Table 5.

### 3.7. Exploratory Cross-Lagged Analysis of Lagged Associations

Exploratory cross-lagged estimates are presented in Table 7. Across 129 transitions from 47 patients, no lagged association was detectable between IL-6 and subsequent VDI (β = 0.04, 95% CI −0.04 to 0.13; *p* = 0.310) or between VDI and subsequent IL-6 (β = −0.05, 95% CI −0.20 to 0.09; *p* = 0.476). Their bootstrap coefficient difference was 0.10 (95% CI −0.10 to 0.30). SOFA predicted subsequent IL-6 (β = 0.18; *p* = 0.007) and VDI (β = 0.19; *p* = 0.009); lactate predicted subsequent SOFA (β = 0.11; *p* = 0.015). Autoregressive coefficients ranged from 0.66 to 0.78 (all *p* < 0.001). Models explained 60%, 70%, 61%, and 53% of subsequent IL-6, VDI, SOFA, and lactate variance. Adjustment for adsorptive therapy yielded reciprocal coefficients of 0.06 (95% CI −0.02 to 0.14) and −0.05 (95% CI −0.19 to 0.09); the IL-6-to-lactate association was no longer significant. Refitting the model separately by interval gave similar reciprocal coefficients for the 94 twelve-hour transitions (IL-6 to VDI β = 0.05, 95% CI −0.06 to 0.16; VDI to IL-6 β = −0.03, 95% CI −0.19 to 0.13) and for the 35 twenty-four-hour transitions (β = 0.02, 95% CI −0.15 to 0.19; β = −0.09, 95% CI −0.33 to 0.15), with correspondingly wider intervals. Given the sample size, these intervals are compatible with small to moderate lagged effects in either direction, and the absence of a detectable association should not be interpreted as evidence that the two processes are unrelated.

### 3.8. Desirability-of-Outcome Ranking

DOOR ranks and win statistics are presented in Supplementary Table S2 and are exploratory. Mean ranks were 3.77 with conventional CRRT, 3.33 with oXiris, and 3.20 with CytoSorb (*p* = 0.223). The pooled comparison included 572 patient pairs, with 181 wins, 56 losses, and 335 ties, yielding a win probability of 0.609 (95% CI 0.492 to 0.725) and a win ratio of 3.23 (95% CI 0.90 to 13.62; *p* = 0.091). The CytoSorb-versus-oXiris win probability was 0.533 (95% CI 0.310 to 0.781). After adjustment for baseline SOFA, the OR for a better rank with adsorptive therapy was 2.74 (95% CI 0.54 to 13.80; *p* = 0.222); the OR per SOFA point was 0.58 (95% CI 0.43 to 0.79; *p* < 0.001). Because the comparator group is heterogeneous, the CytoSorb group is very small, and treatment allocation was not random, these win statistics are descriptive summaries and are not presented as efficacy estimates.

### 3.9. Penalised 24 H Landmark Prediction Models for 28-Day Survival

Prediction models and their performance are presented in Supplementary Table S3. The baseline SOFA-and-age model had an apparent AUC of 0.888 and an optimism-corrected AUC of 0.882. Adding favourable haemodynamic status yielded an adjusted OR of 3.21 (95% CI 0.51 to 20.32; *p* = 0.199) and a corrected AUC of 0.866. The full model M3, which has five predictors and 12 events, falls below the recommended events-per-variable ratio and is reported only for completeness; it had a corrected AUC of 0.842; neither the IL-6 log-ratio (*p* = 0.633) nor adsorptive device (*p* = 0.242) was significant. Corrected Brier scores increased from 0.125 to 0.144 across models, while Nagelkerke R^2^ ranged from 0.50 to 0.56. In a sensitivity model restricted to the 35 vasopressor-dependent patients, haemodynamic response by the VDI criterion adjusted for baseline SOFA and age had an OR of 5.12 (95% CI 0.71 to 36.9; *p* = 0.108).

Recalibration of the original data yielded intercepts of 0.06, 0.04, and 0.05 and slopes of 1.15, 1.19, and 1.31 for M1–M3. In decision-curve analysis, all models had higher estimated net benefit than the all-positive and all-negative strategies across thresholds of 5–70%. Net benefit for M1 was 0.156 at a 15% threshold; values at 30% were 0.167, 0.128, and 0.149 for M1–M3. The curves crossed, with between-model differences no greater than approximately 0.06 (Supplementary Table S3).

## 4. Discussion

### 4.1. Interpretation and Comparison with Previous Studies

In this retrospective cohort of patients with sepsis-associated acute kidney injury requiring CRRT, the main findings were differences in selected unadjusted haemodynamic and organ-dysfunction trajectories that were attenuated after adjustment for baseline values, no statistically demonstrable survival benefit, and no detectable lagged association between IL-6 and vasopressor dependency in either direction. The strongest univariable response association involved haemodynamic status, but its addition to baseline SOFA and age did not improve optimism-corrected discrimination. These observations distinguish changes during treatment from evidence that the treatment caused recovery. They are consistent with the uncertainty in randomised studies and the meta-analysis already described [7,9], while the five-patient CytoSorb group, the heterogeneous comparator and confounding by indication—evident in the younger age and higher norepinephrine, VDI, IL-6, and procalcitonin values of the CytoSorb group—preclude any conclusion about device effectiveness. The larger fall in VDI observed with CytoSorb cannot be attributed to the device on the basis of these data.

Extracorporeal devices differ in their adsorption mechanisms and target molecules [34,35]. The in vitro comparison by Malard et al. found broadly similar removal of many inflammatory mediators with oXiris and CytoSorb, while oXiris also removed endotoxin [35]. It therefore does not establish that CytoSorb has uniformly superior cytokine removal. In the present cohort, the larger decline in the CytoSorb group occurred alongside much higher baseline IL-6 and VDI values. A greater opportunity for absolute improvement, regression to the mean, and clinician selection are plausible explanations in addition to a device effect. These alternatives cannot be separated by the available observational comparison, and the attenuation of the CytoSorb contrast after adjustment for baseline VDI (Table 4) supports their importance. Systematic reviews of oXiris and of CytoSorb likewise report favourable physiological signals in observational data but rate the certainty of evidence as low or very low and remain inconclusive regarding mortality [10,11,12,13].

Previous oXiris case series reported reductions in norepinephrine requirements and lactate [36], whereas propensity-weighted or matched CytoSorb cohorts described favourable haemodynamic or mortality associations [37,38]. A refractory-shock series and a small randomised pilot trial produced differing clinical signals [39,40]. Such variation may reflect differences in baseline severity, timing, patient selection, and concurrent treatment. Broman et al. demonstrated biochemical changes with oXiris in a small crossover study [6], while Schädler et al. observed IL-6 extraction across CytoSorb without a significant between-group reduction in systemic concentrations [7]. Device extraction, a falling circulating concentration, and a better clinical outcome are therefore separate questions. The earlier ECMO-associated cohorts provide related physiological observations [17,18], but differences in respiratory support and treatment exposure limit direct comparison. Those cohorts were assembled at the same centre, over an overlapping calendar period and by an overlapping group of investigators; however, they comprised exclusively patients supported with ECMO, who were not eligible for the present study, and no patient in the present cohort was included in either of those publications.

The absence of detectable reciprocal lagged associations indicates only that, at the sampled intervals and with 47 patients, IL-6 changes were not measurably associated with subsequent vasopressor changes in this cohort; the confidence intervals are compatible with small to moderate effects in either direction. It does not establish that cytokines and vascular dysfunction are mechanistically independent. Circulating concentrations can change through production, redistribution, endogenous clearance and extracorporeal removal; the present study measured concentrations rather than trans-device clearance. The absence of a clear clinical benefit from high-volume haemofiltration [41] and the heterogeneity of sepsis phenotypes [42] further support evaluating these processes in their clinical context. Some patients showed haemodynamic improvement without a large IL-6 decline, or the reverse, but the small phenotype groups cannot support any biological classification of response.

SOFA predicted subsequent IL-6 and VDI, which is compatible with a relationship between overall illness severity and both trajectories. However, the cardiovascular component of SOFA already incorporates blood pressure and vasoactive support [24]. Its association with VDI is therefore partly vulnerable to overlap between the measures, rather than necessarily indicating a distinct upstream mechanism. Similarly, VDI includes MAP in its denominator, so the inverse correlation between changes in VDI and MAP is partly mathematical. These dependencies should be considered when interpreting the apparent haemodynamic and organ-function cluster. A future analysis excluding the cardiovascular SOFA component could help assess the extent of this overlap.

### 4.2. Clinical and Methodological Implications

Haemodynamic response is a clinically interpretable candidate endpoint because it combines changes in vasoactive support with the achieved arterial pressure. Nevertheless, the VIS weights cited here originate from a different clinical setting [26], and neither the VDI nor its response threshold has been externally validated as a surrogate for survival in this population. The favourable univariable association also weakened after adjustment for baseline SOFA and age. It should therefore motivate prospective evaluation of response criteria, rather than a treatment-selection or stopping rule. These findings do not justify choosing a cartridge, continuing adsorption, or escalating therapy solely because a cytokine concentration falls.

DOOR complements mortality by ordering outcomes according to clinically defined priorities [20,30]. In this cohort, however, most patients received the death rank, and many pairwise comparisons were ties. The win probability includes half of those ties; it is not simply the probability of a strictly better outcome. Its confidence interval included the null value, and the baseline-adjusted estimate remained imprecise. Accordingly, and because the comparator group was not uniformly non-adsorptive, the analysis provides an exploratory summary without demonstrating superiority of adsorptive therapy, and the win statistics are reported in Supplementary Table S2 rather than as principal results. Reporting each component alongside the composite remains necessary so that differences in survival, vasopressor liberation, and organ dysfunction remain visible.

The prediction analyses illustrate the distinction between association, discrimination, and clinical usefulness. Firth penalisation reduces some small-sample estimation problems but cannot create information absent from the cohort [21]. Bootstrap correction showed that adding response variables increased apparent performance without improving corrected discrimination or prediction error, and the model containing five predictors for 12 events is reported only in Supplementary Table S3 as an illustration of this overfitting. Internal validation is not external validation, and the baseline model should not be adopted as a bedside prognostic tool on these results alone [33]. Decision curves additionally require a specified action and an appropriate threshold [32]. Here, the analyses are exploratory; a higher estimated net benefit does not by itself identify a clinical decision that improves patient outcomes.

### 4.3. Study Limitations

The retrospective, single-centre design, small sample, and only 12 survivors limit precision, model complexity, and generalisability. The CytoSorb group contained five patients, making device-specific estimates particularly unstable; all CytoSorb comparisons should be regarded as descriptive. Treatment selection was determined by clinical practice and device availability, producing confounding by indication with baseline inflammatory, vasopressor, age, and renal differences between groups; the three-group comparisons and subgroup analyses are therefore underpowered, and the observed trajectory differences cannot establish device effects. Adjustment for SOFA cannot exclude residual confounding, and the comparisons should remain observational associations [22]. The cohort was predominantly pneumonic and severely ill, so the findings may not transfer to other infection sources or less severe sepsis. Concurrent antimicrobial treatment, source control, fluid management, and other supportive interventions could also influence the observed trajectories.

The principal mixed models specified time as a linear term; because the observed trajectories were visibly non-linear, the reported slopes are average rates of change over 48 h rather than descriptions of trajectory shape, although the categorical-time sensitivity analysis led to the same conclusions. The longitudinal analysis pooled two 12 h transitions and one 24 h transition. Interval indicators allow different intercepts but do not establish that the lagged coefficients are identical across intervals; interval-specific models gave similar point estimates with wider intervals, but with 47 patients, none of these models can distinguish a true absence of association from an undetected one. Moreover, clustered standard errors account for repeated observations without separating stable between-patient differences from within-patient change, a recognised limitation of conventional cross-lagged models [19]. The short observation window, sparse sampling and limited cohort size may therefore obscure faster, slower, or heterogeneous temporal relationships. Null coefficients should not be interpreted as evidence that the two processes are unrelated.

Missingness also warrants caution: ten patients lacked a complete usable assessment at 48 h, and nine of them died. Available-case trajectories and the mixed-model missing-at-random assumption may therefore be influenced by informative loss of observations; the completer and worst-case sensitivity analyses gave similar patterns but cannot exclude bias from early deaths, which preferentially removed conventional-group patients from the 48 h trajectories. The response analysis therefore treats death before response as a competing event, although with 36 patients the cumulative-incidence estimates remain imprecise. Analyses incorporating measurements at 24 h are conditional on availability at that time and should be interpreted as landmark assessments rather than predictions available at CRRT initiation.

Assay ceilings, harmonisation of vasopressin units and exclusion of implausible recorded values introduce additional measurement uncertainty. A circulating IL-6 decline cannot quantify extracorporeal cytokine clearance without inlet, outlet and flow measurements. The conventional group included different filters and two CRRT platforms, and five patients received PMMA membranes, which adsorb cytokines [14,15,16]; although the principal results were similar after excluding these patients (Table 4), the comparator should not be regarded as a uniform non-adsorptive control, and the study is framed as a comparison of device strategies rather than of adsorptive versus non-adsorptive CRRT. Anticoagulation and circuit duration also varied. Multiple secondary and subgroup comparisons were performed; after Benjamini–Hochberg adjustment, none of the between-group outcome differences remained significant, and isolated nominal findings are exploratory. Finally, renal recovery, dialysis dependence, longer-term function, and a dedicated comparative safety assessment were not available as outcomes.

### 4.4. Future Research Directions

A prospective multicentre comparison should define a common treatment start, device exposure, and replacement schedule, standardise haemodynamic measurements, and record concurrent care. Repeated cytokine and haemodynamic sampling at consistent intervals would permit clearer separation of treatment exposure from disease evolution. Where feasible, trans-device measurements should distinguish extraction from systemic concentration changes. Larger cohorts could support models separating within-patient change from stable patient characteristics and sensitivity analyses excluding the cardiovascular SOFA component.

Future trials should prioritise patient-centred outcomes, including survival, kidney recovery, time free of organ support and adverse events, with haemodynamic response and cytokine kinetics as complementary measures. A clinically agreed ordinal endpoint could retain useful information, provided that its components and handling of missing follow-up are specified in advance. Baseline vasopressor burden and hyperlactataemia are possible stratification variables, but the present non-significant subgroup interactions do not establish enrichment criteria. Independent validation is needed before either a response threshold or a prognostic model informs treatment decisions.

## 5. Conclusions

In this exploratory single-centre cohort of patients with sepsis-associated acute kidney injury receiving continuous renal replacement therapy, selected haemodynamic and organ-dysfunction trajectories differed between treatment strategies in unadjusted analyses, but these differences were attenuated after adjustment for baseline values and, given confounding by indication and the five-patient CytoSorb group, cannot be attributed to the devices; no survival advantage was demonstrable. No lagged association between cytokine changes and vasopressor dependency was detectable at the sampled intervals, which does not exclude such a relationship. Early haemodynamic status was associated with survival in univariable analysis, but did not improve internally corrected prediction beyond baseline severity and age. These findings support prospective evaluation of haemodynamic response alongside patient-centred outcomes and caution against treating biomarker reduction as evidence of clinical benefit. Larger comparative studies are required before the findings can guide device selection.

## Figures and Tables

**Figure 1:**
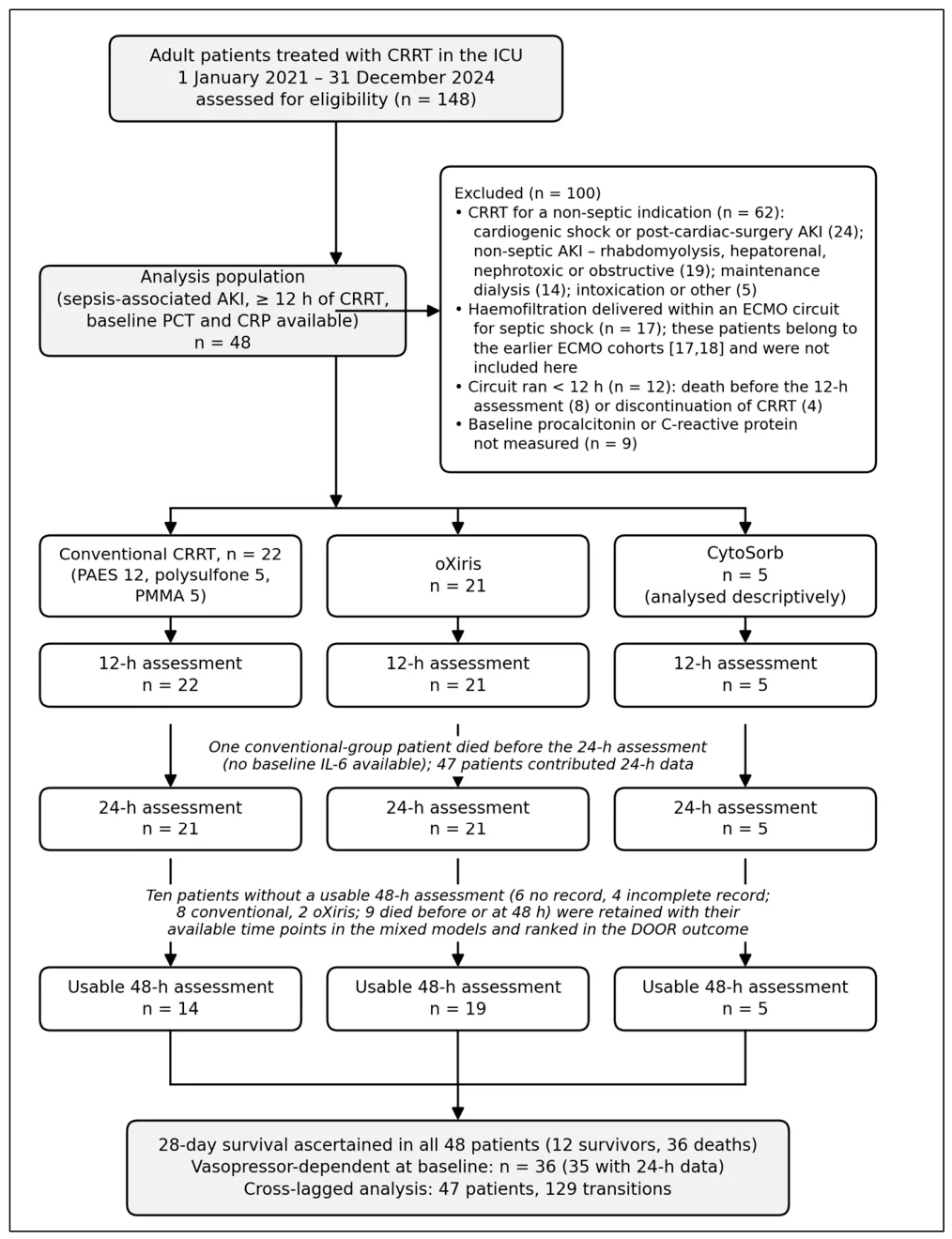
STROBE-style flow diagram of patient screening, exclusion and follow-up. Of 148 adult patients treated with CRRT in the intensive care unit between 1 January 2021 and 31 December 2024, 100 were excluded for the reasons shown, and 48 constituted the analysis population (conventional CRRT 22, oXiris 21, CytoSorb 5). The numbers of patients contributing at the 12 h, 24 h, and 48 h assessments are shown by treatment strategy; the ten patients without a usable 48 h assessment were retained with their available time points in the mixed models and ranked in the DOOR outcome. Patients treated with haemofiltration integrated into an ECMO circuit were reported in earlier cohorts [17,18] and do not overlap with the present study. Abbreviations: AKI, acute kidney injury; CRP, C-reactive protein; CRRT, continuous renal replacement therapy; DOOR, desirability of outcome ranking; ECMO, extracorporeal membrane oxygenation; ICU, intensive care unit; IL-6, interleukin-6; PAES, polyarylethersulfone; PCT, procalcitonin; PMMA, polymethylmethacrylate; STROBE, Strengthening the Reporting of Observational Studies in Epidemiology.

**Figure 2:**
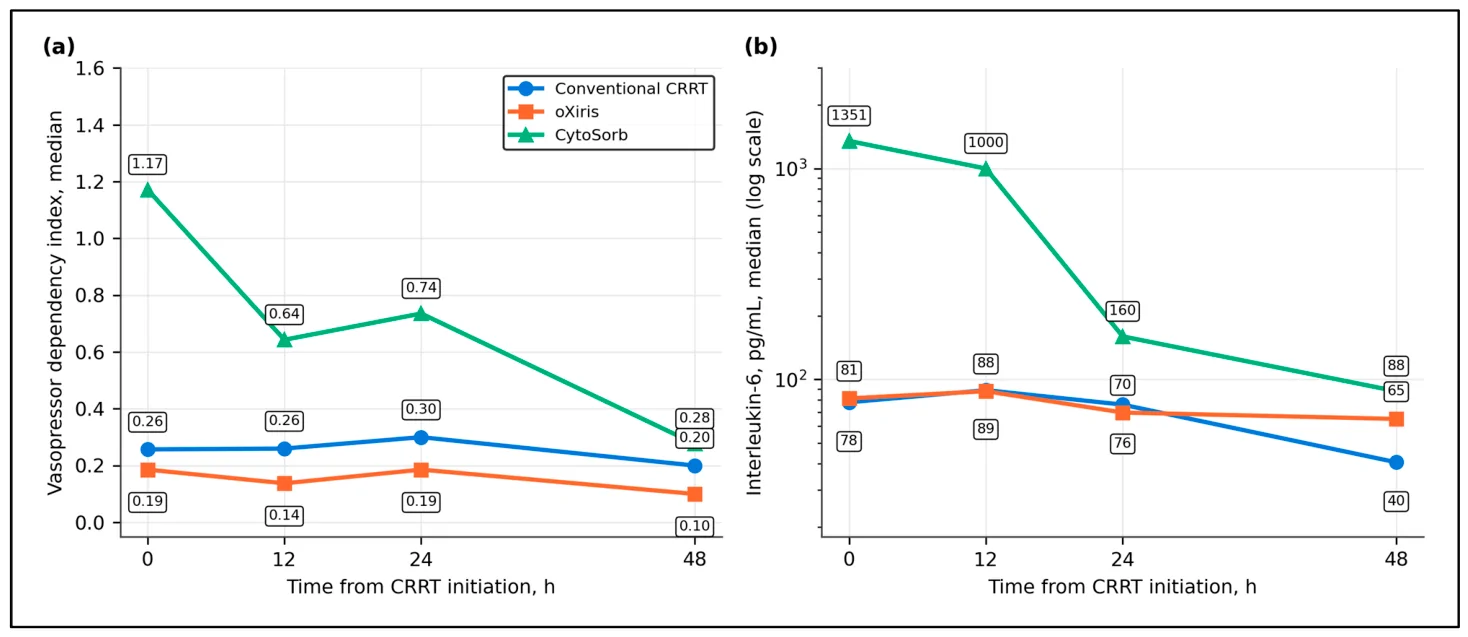
Median trajectories of (**a**) the vasopressor dependency index and (**b**) interleukin-6 (logarithmic axis) from CRRT initiation to 48 h according to treatment strategy. Labels show median values; numbers of patients contributing at 0, 12, 24, and 48 h were 22, 22, 21, and 14 (conventional), 21, 21, 21, and 19 (oXiris), and 5, 5, 5, and 5 (CytoSorb) for VDI, with one fewer conventional patient at 0 and 12 h and one fewer CytoSorb patient at 48 h for IL-6.

**Figure 3:**
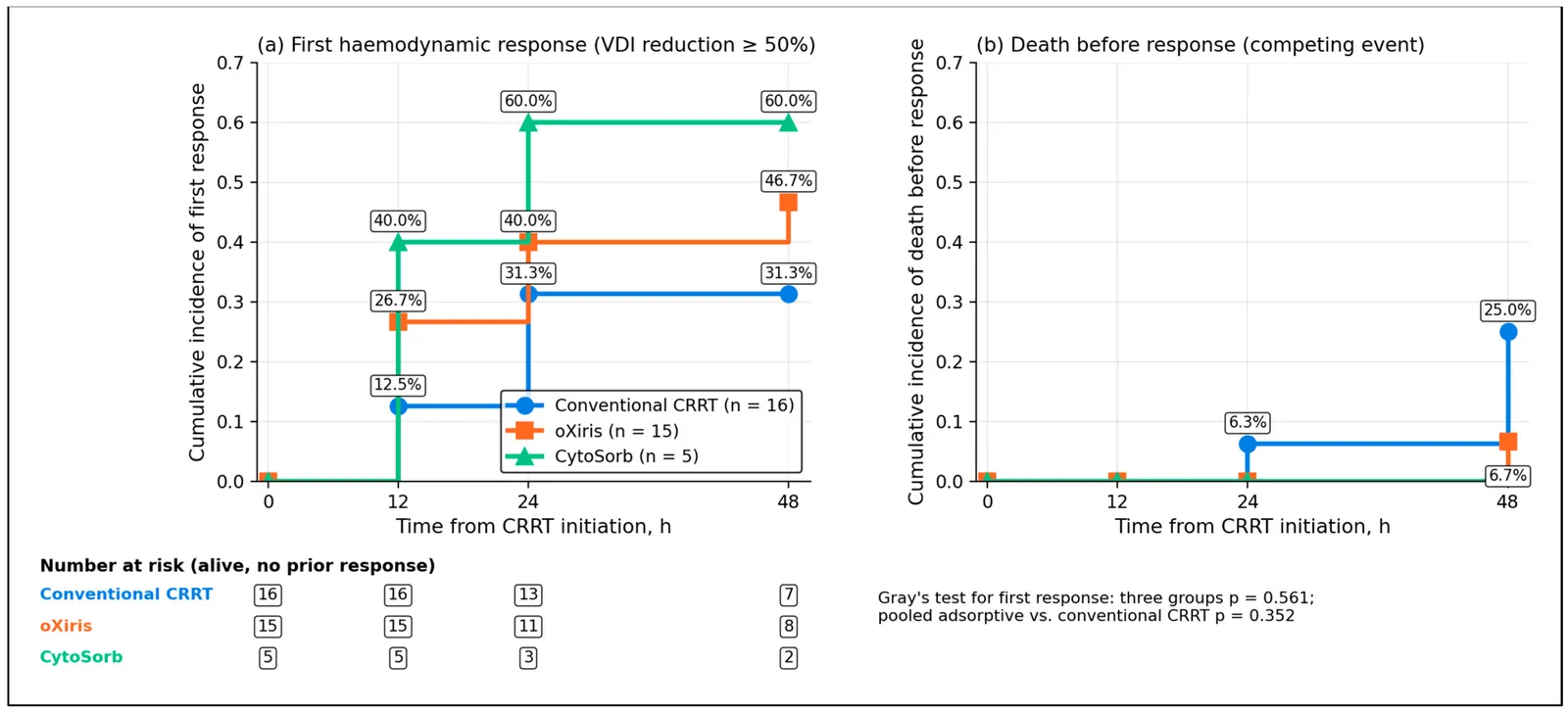
Cumulative incidence of (**a**) a first reduction in the vasopressor dependency index (VDI) of at least 50% and (**b**) death before haemodynamic response, in vasopressor-dependent patients (*n* = 36) at 12, 24 and 48 h after CRRT initiation, by treatment strategy (discrete-time Aalen–Johansen estimates with death treated as a competing event; Gray’s test for first response: three groups *p* = 0.561, pooled adsorptive versus conventional *p* = 0.352). Numbers at risk (alive without prior response) are shown below panel (**a**).

**Figure 4:**
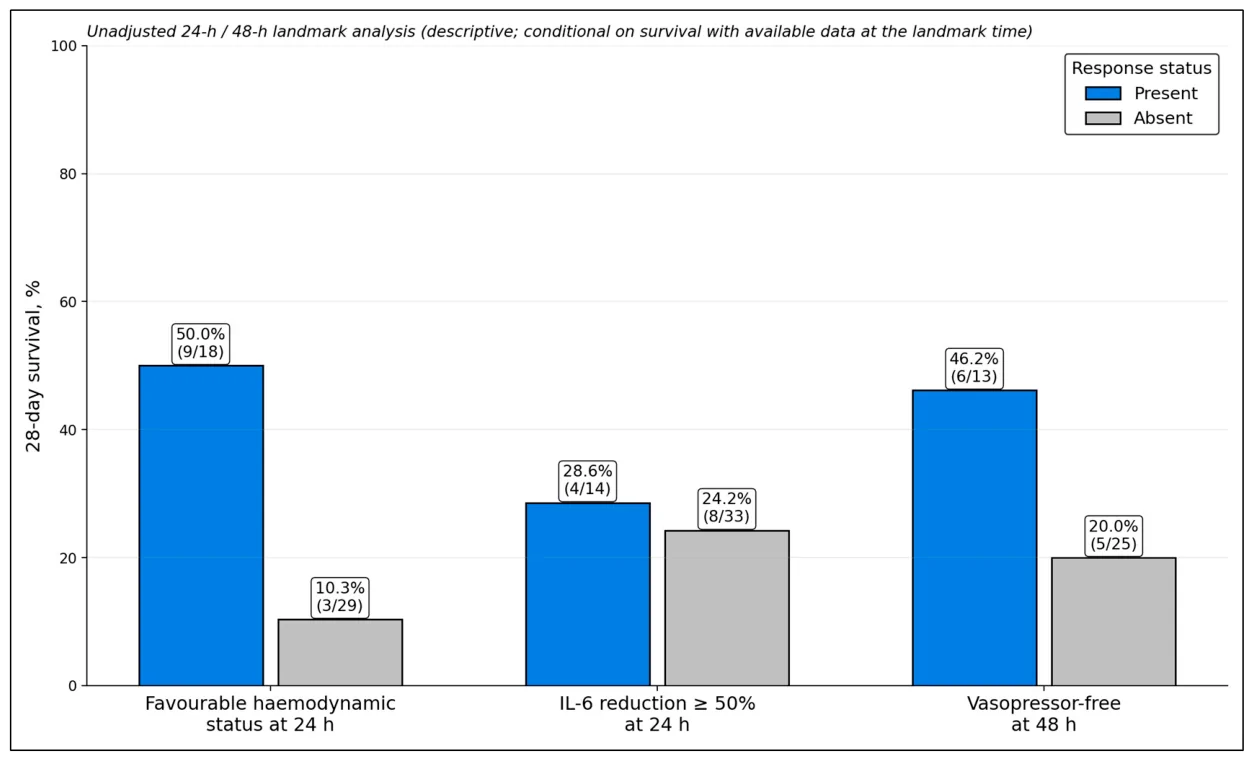
Unadjusted 24 h/48 h landmark (descriptive) analysis of twenty-eight-day survival according to the presence or absence of three early-response criteria: favourable haemodynamic status at 24 h (VDI reduction ≥ 50% in vasopressor-dependent patients or persistence of a vasopressor-free state), interleukin-6 reduction ≥ 50% at 24 h, and vasopressor-free status at 48 h. Labels give the percentage and the number of survivors/patients with available data. Each comparison is conditional on survival with available data at the landmark time point and does not represent a baseline survival predictor.

**Figure 5:**
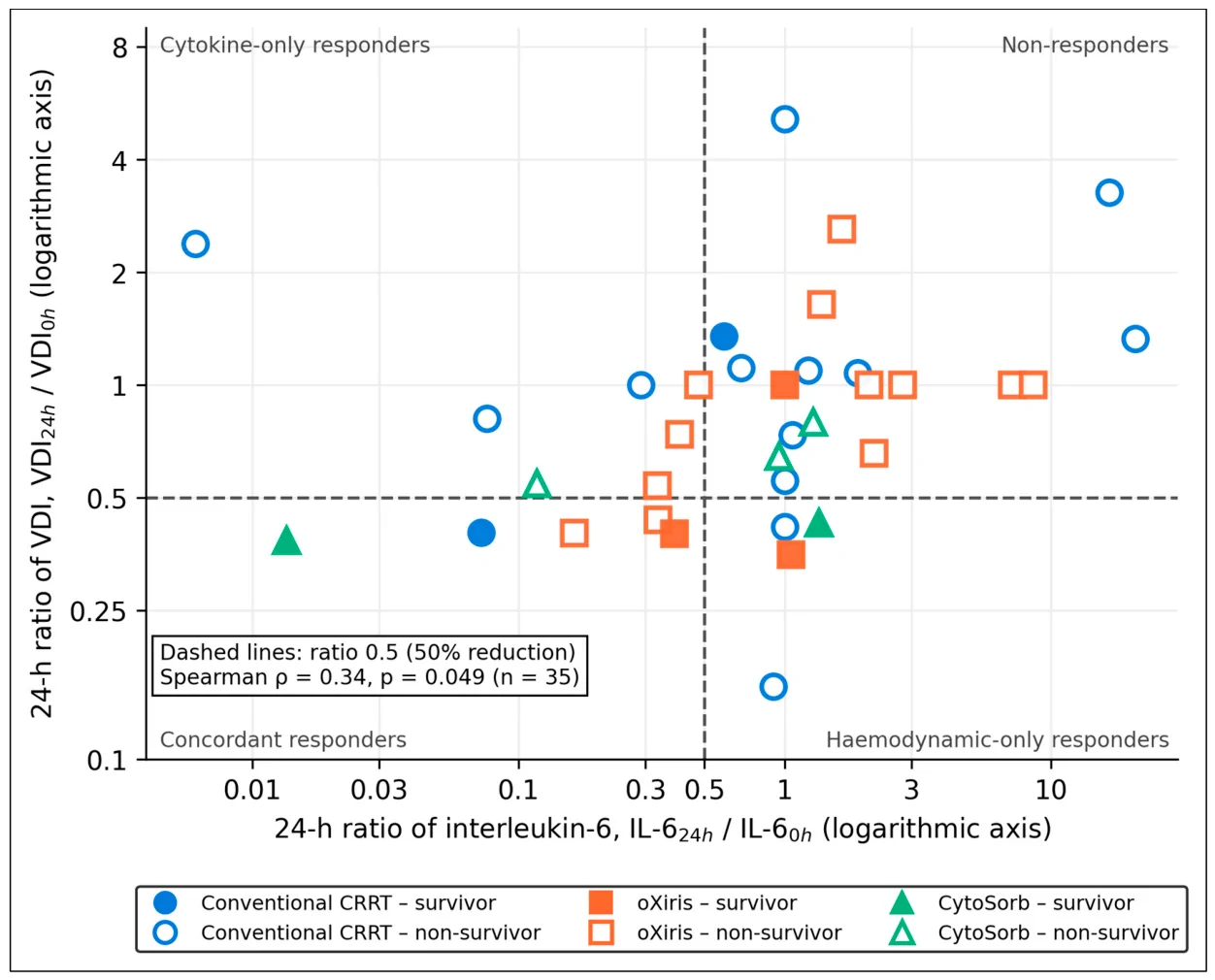
Cytokine and vasopressor response map in vasopressor-dependent patients (*n* = 35): 24 h ratio of interleukin-6 (IL-6 at 24 h divided by IL-6 at baseline; x-axis) versus 24 h ratio of the vasopressor dependency index (VDI at 24 h divided by VDI at baseline; y-axis), both plotted on logarithmic axes without any offset. Dashed lines mark a ratio of 0.5, i.e., exactly the 50% reduction used to define response in Table 2 and Table 5, indicating four response phenotypes (lower left, concordant responders; upper left, cytokine-only responders; lower right, haemodynamic-only responders; upper right, non-responders); colours and symbols denote treatment strategy, filled symbols denote 28-day survivors. Because the displayed threshold corresponds exactly to the raw response definition, all nine haemodynamic responders lie below the horizontal dashed line, and no exception is required; the offset of 0.05 used for log transformation in the mixed models is not applied in this figure. Spearman ρ refers to the correlation between the logarithms of the two ratios.

**Table 1:** Baseline demographic, clinical, renal, infection, circuit and laboratory characteristics of the cohort according to treatment strategy.

Characteristic	Conventional CRRT (*n* = 22)	oXiris (*n* = 21)	CytoSorb (*n* = 5)	All Patients (*n* = 48)	*p*
Age, years	68.0 (60.2–73.5)	64.0 (55.0–75.0)	54.0 (46.0–57.0)	65.5 (55.8–74.0)	0.070
Male sex	13 (59.1)	12 (57.1)	3 (60.0)	28 (58.3)	1.000
Body mass index, kg/m^2^	27.6 (24.3–33.4)	31.2 (26.0–33.3)	26.9 (25.9–30.9)	29.4 (25.3–33.2)	0.489
Arterial hypertension	14 (63.6)	16 (76.2)	3 (60.0)	33 (68.8)	0.601
Diabetes mellitus	5 (22.7)	8 (38.1)	1 (20.0)	14 (29.2)	0.527
Septic shock at CRRT initiation	17 (77.3)	16 (76.2)	5 (100.0)	38 (79.2)	0.558
ARDS	7 (31.8)	6 (28.6)	3 (60.0)	16 (33.3)	0.457
SARS-CoV-2 infection	3 (13.6)	3 (14.3)	0 (0.0)	6 (12.5)	0.743
Pneumonia as infection source	13 (59.1)	13 (61.9)	3 (60.0)	29 (60.4)	0.968
Intra-abdominal infection source	5 (22.7)	4 (19.0)	1 (20.0)	10 (20.8)	1.000
Urinary, skin/soft-tissue or other source	4 (18.2)	4 (19.0)	1 (20.0)	9 (18.8)	1.000
APACHE II score	23.0 (20.2–30.5)	22.0 (16.0–26.0)	26.0 (15.0–33.0)	23.0 (15.8–28.2)	0.622
SOFA score at baseline	12.0 (10.2–13.8)	12.0 (9.0–13.0)	12.0 (9.0–16.0)	12.0 (9.8–13.0)	0.477
KDIGO stage 3 acute kidney injury	19 (86.4)	17 (81.0)	4 (80.0)	40 (83.3)	0.851
Time from ICU admission to CRRT, days	2.0 (1.0–4.0)	2.0 (1.0–3.0)	1.0 (1.0–2.0)	2.0 (1.0–3.2)	0.412
Vasopressor support at baseline	16 (72.7)	15 (71.4)	5 (100.0)	36 (75.0)	0.454
Prescribed effluent dose, mL/kg/h	34.5 (32.2–35.0)	34.0 (32.0–35.0)	35.0 (35.0–35.0)	34.5 (32.8–35.0)	0.317
Circuit duration, h	34.0 (26.5–51.5)	72.0 (29.0–72.0)	48.0 (36.0–72.0)	48.0 (28.0–72.0)	0.115
PMMA haemofilter	5 (22.7)	0 (0.0)	0 (0.0)	5 (10.4)	—
CVVHDF modality	16 (72.7)	17 (81.0)	4 (80.0)	37 (77.1)	0.803
Unfractionated heparin anticoagulation	18 (81.8)	19 (90.5)	5 (100.0)	42 (87.5)	0.634
***Haemodynamic and laboratory parameters at CRRT initiation***					
Mean arterial pressure, mmHg	75.0 (65.0–83.0)	70.0 (68.0–83.0)	65.0 (65.0–70.0)	70.0 (65.0–83.0)	0.304
Norepinephrine, µg/kg/min	0.18 (0.00–0.65)	0.14 (0.00–0.38)	0.82 (0.32–1.06)	0.17 (0.01–0.50)	0.064
Vasopressor dependency index	0.26 (0.00–0.88)	0.19 (0.00–0.54)	1.17 (0.46–1.66)	0.26 (0.01–0.69)	0.063
Lactate, mmol/L	2.0 (1.5–3.2)	1.7 (1.4–2.4)	3.7 (2.6–3.8)	2.0 (1.5–3.1)	0.160
Interleukin-6, pg/mL	78.0 (25.0–233.0)	81.3 (29.0–165.0)	1351.0 (59.0–1897.0)	81.3 (28.0–260.5)	0.245
Procalcitonin, ng/mL	3.0 (0.9–9.3)	4.3 (1.8–12.2)	100.0 (31.6–100.0)	3.7 (1.5–17.8)	0.030
C-reactive protein, mg/L	111.0 (32.4–195.5)	163.0 (102.0–267.0)	318.6 (99.9–462.0)	155.5 (76.7–269.2)	0.104
Ferritin, ng/mL	877.5 (364.5–2584.2)	1488.0 (483.0–4159.0)	1758.0 (1029.0–4393.0)	1358.0 (416.2–2773.2)	0.419
Creatinine, mg/dL	3.3 (1.8–5.0)	3.5 (2.4–4.5)	2.1 (0.5–3.4)	3.3 (2.0–4.4)	0.211
Urea, mg/dL	205.0 (116.2–270.5)	162.0 (119.0–192.0)	83.0 (81.0–100.5)	170.0 (105.9–224.2)	0.018

Values are median (interquartile range) or *n* (%). *p*-values from the Kruskal–Wallis test for continuous variables and Fisher’s exact test (Monte-Carlo extension for 3 × 2 tables) for categorical variables. Vasoactive-inotropic score = 100 × norepinephrine (µg/kg/min) + 10,000 × vasopressin (U/kg/min); vasopressor dependency index = vasoactive-inotropic score/mean arterial pressure. The PMMA row is shown by design and was not tested. Abbreviations: APACHE II, Acute Physiology and Chronic Health Evaluation II; ARDS, acute respiratory distress syndrome; BMI, body mass index; CRRT, continuous renal replacement therapy; CVVHDF, continuous veno-venous haemodiafiltration; ICU, intensive care unit; KDIGO, Kidney Disease: Improving Global Outcomes; PMMA, polymethylmethacrylate; SARS-CoV-2, severe acute respiratory syndrome coronavirus 2; SOFA, Sequential Organ Failure Assessment.

**Table 2:** Primary and secondary outcomes according to treatment strategy.

Outcome	Conventional CRRT	oXiris	CytoSorb	All Patients	*p*
28-day survival	3 (13.6)	7 (33.3)	2 (40.0)	12 (25.0)	0.240
Haemodynamic response at 24 h (VDI reduction ≥ 50%) ^1^	3 (20.0)	4 (26.7)	2 (40.0)	9 (25.7)	0.866
Vasopressor-free at 48 h ^2^	6 (42.9)	6 (31.6)	1 (20.0)	13 (34.2)	0.713
Absolute change in VDI, 0–24 h ^1^	0.05 (−0.18 to 0.11)	−0.01 (−0.19 to 0.00)	−0.44 (−0.46 to −0.38)	−0.06 (−0.34 to 0.05)	0.018
Change in norepinephrine-equivalent dose, 0–24 h, µg/kg/min ^1^	0.00 (−0.10 to 0.08)	−0.02 (−0.12 to 0.01)	−0.35 (−0.48 to −0.20)	−0.03 (−0.15 to 0.04)	0.021
Change in MAP, 0–24 h, mmHg	3.0 (−5.0 to 10.0)	5.0 (−2.0 to 12.0)	8.0 (0.0 to 12.0)	5.0 (−3.0 to 11.0)	0.412
Relative change in IL-6, 0–24 h, %	−2.6 (−42.9–22.2)	5.5 (−57.6–107.5)	−4.6 (−88.2–27.7)	0.0 (−58.6–50.8)	0.499
IL-6 reduction ≥ 50% at 24 h	5 (23.8)	7 (33.3)	2 (40.0)	14 (29.8)	0.737
Change in lactate, 0–24 h, mmol/L	0.0 (−0.5–0.8)	−0.4 (−0.8 to −0.1)	−0.6 (−1.1 to −0.5)	−0.4 (−0.7–0.2)	0.035
Change in SOFA, 0–48 h, points	−2.5 (−4.8 to −1.0)	−2.0 (−3.0–0.2)	−4.0 (−4.0 to −4.0)	−2.0 (−4.0–0.0)	0.047
SOFA improvement ≥ 2 points at 48 h	9 (64.3)	11 (55.0)	4 (100.0)	24 (63.2)	0.318

Values are *n* (%) or median (interquartile range). Denominators are 22, 21, 5 and 48 patients for 28-day survival; 21, 21, 5 and 47 for 24 h variables; ^1^ 15, 15, 5 and 35 vasopressor-dependent patients with 24 h data; ^2^ 14, 19, 5 and 38 patients with 48 h data (14, 20, 4 and 38 for the SOFA rows). *p*-values from Fisher’s exact test (Monte-Carlo extension) or the Kruskal–Wallis test; after Benjamini–Hochberg adjustment across the eleven outcomes in this table, all adjusted *p*-values were ≥0.116. Norepinephrine-equivalent dose = norepinephrine (µg/kg/min) + 2.5 × vasopressin (U/min). Unadjusted between-group differences do not account for the baseline imbalances shown in Table 1. Abbreviations: CRRT, continuous renal replacement therapy; IL-6, interleukin-6; MAP, mean arterial pressure; SOFA, Sequential Organ Failure Assessment; VDI, vasopressor dependency index.

**Table 3:** Linear mixed-effects models of haemodynamic, inflammatory, and organ-dysfunction trajectories from 0 to 48 h according to treatment strategy.

Variable (Scale)	Conventional CRRT Slope per 24 h (95% CI)	oXiris Slope per 24 h (95% CI)	CytoSorb Slope per 24 h (95% CI)	*p* for Group × Time Interaction
Vasopressor dependency index (log)	0.03 (−0.16 to 0.22)	−0.02 (−0.19 to 0.15)	−0.44 (−0.78 to −0.11)	0.050
Norepinephrine-equivalent dose (log)	0.02 (−0.15 to 0.19)	−0.04 (−0.20 to 0.12)	−0.38 (−0.70 to −0.06)	0.078
Mean arterial pressure, mmHg	2.5 (−1.4 to 6.3)	2.7 (−0.7 to 6.1)	2.0 (−4.9 to 8.9)	0.984
Interleukin-6 (log)	−0.31 (−0.64 to 0.02)	0.09 (−0.20 to 0.39)	−0.75 (−1.39 to −0.11)	0.034
Procalcitonin (log)	−0.29 (−0.47 to −0.10)	−0.45 (−0.61 to −0.29)	−0.52 (−0.88 to −0.17)	0.322
Lactate, mmol/L	0.28 (−0.05 to 0.62)	−0.13 (−0.43 to 0.16)	−0.30 (−0.88 to 0.28)	0.110
SOFA score, points	−1.05 (−1.67 to −0.42)	−0.13 (−0.68 to 0.42)	−1.88 (−3.09 to −0.68)	0.012

Slopes are fixed-effect estimates per 24 h (95% CI) from random-intercept linear mixed-effects models with a group × time interaction, based on 178–182 observations from 47 to 48 patients (48, 48, 47, and 38 patients at 0, 12, 24, and 48 h for haemodynamic variables and SOFA; 47, 47, 47, and 37 for IL-6); the interaction *p*-value is from the likelihood-ratio test of nested maximum-likelihood models. VDI was log-transformed after adding 0.05 and the norepinephrine-equivalent dose after adding 0.01, so that the slopes for these two variables describe change in log(VDI + 0.05) and log(norepinephrine-equivalent dose + 0.01) and their exponentiated values are ratios of the offset-transformed variables per 24 h, not of the unmodified variables; IL-6 and procalcitonin were log-transformed without an offset. Time was modelled as a linear term; the slopes summarise the average rate of change over 0–48 h and do not imply that the trajectories were linear (see the categorical-time sensitivity analysis in Section 3.3 and Table 4). Abbreviations: CI, confidence interval; CRRT, continuous renal replacement therapy; IL-6, interleukin-6; SOFA, Sequential Organ Failure Assessment; VDI, vasopressor dependency index.

**Table 5:** Univariable associations of baseline variables and 24 h/48 h landmark early-response variables with 28-day survival.

Variable	Survivors (*n* = 12)	Non-Survivors (*n* = 36)	OR for Survival (95% CI) †	*p*
Age, years	61.0 (49.8–67.2)	67.0 (57.0–75.0)	0.57 (0.29 to 1.11)	0.084
APACHE II score	19.5 (15.0–25.0)	23.0 (16.8–29.5)	0.48 (0.22 to 1.07)	0.065
SOFA score at baseline	9.0 (6.0–11.2)	12.0 (11.8–14.0)	0.27 (0.11 to 0.65)	0.002
Baseline norepinephrine, µg/kg/min	0.07 (0.00–0.21)	0.22 (0.08–0.61)	0.59 (0.25 to 1.39)	0.066
Baseline lactate, mmol/L	1.5 (1.3–1.9)	2.2 (1.7–3.3)	0.54 (0.20 to 1.46)	0.058
Baseline IL-6, pg/mL (log-transformed)	4.8 (4.1–6.3)	4.1 (3.3–5.3)	1.33 (0.69 to 2.54)	0.341
Baseline C-reactive protein, mg/L	251.5 (83.0–315.1)	139.5 (69.8–201.2)	1.50 (0.79 to 2.84)	0.143
Baseline urea, mg/dL	109.5 (89.8–171.2)	186.5 (117.5–247.8)	0.39 (0.16 to 0.92)	0.012
Adsorptive device (oXiris or CytoSorb)	9 (75.0)	17 (47.2)	3.02 (0.73 to 12.45)	0.180
Haemodynamic response at 24 h (VDI reduction ≥ 50%) ^1^	5 (71.4)	4 (14.3)	11.98 (1.85 to 77.43)	0.006
Favourable haemodynamic status at 24 h ^2^	9 (75.0)	9 (25.7)	7.57 (1.75 to 32.69)	0.005
IL-6 reduction ≥ 50% at 24 h	4 (33.3)	10 (28.6)	1.29 (0.32 to 5.15)	0.731
Vasopressor-free at 48 h	6 (54.5)	7 (25.9)	3.23 (0.76 to 13.80)	0.135

Values are median (interquartile range) or *n* (%). Baseline IL-6 was available in 35 non-survivors; 24 h variables in 12 survivors and 35 non-survivors; ^1^ 7 survivors and 28 non-survivors who were vasopressor-dependent at baseline; vasopressor-free status at 48 h in 11 survivors and 27 non-survivors. *p*-values from the Mann–Whitney U or Fisher’s exact test. † Odds ratios for survival per one standard deviation (continuous variables) or for the category versus its absence, from Firth-penalised logistic regression with Wald 95% CIs. ^2^ VDI reduction ≥ 50% in vasopressor-dependent patients or persistence of a vasopressor-free state in others. Abbreviations: APACHE II, Acute Physiology and Chronic Health Evaluation II; CI, confidence interval; IL-6, interleukin-6; OR, odds ratio; SD, standard deviation; SOFA, Sequential Organ Failure Assessment; VDI, vasopressor dependency index. Early-response variables (^1^, ^2^ and vasopressor-free status at 48 h) are landmark analyses conditional on survival with available data at 24 h or 48 h; they are not baseline predictors of survival.

**Table 6:** Spearman rank correlations between 24 h changes in haemodynamic, inflammatory, and metabolic variables and the 48 h change in SOFA score.

Variable	Δ VDI 0–24 h	Δ MAP 0–24 h	Δ IL-6 0–24 h, %	Δ CRP 0–24 h, %	Δ Lactate 0–24 h	Δ SOFA 0–48 h
Δ VDI 0–24 h	—	−0.35 (0.040)	0.28 (0.102)	−0.17 (0.322)	0.09 (0.601)	0.36 (0.060)
Δ MAP 0–24 h	−0.35 (0.040)	—	−0.24 (0.097)	−0.03 (0.867)	−0.13 (0.383)	−0.33 (0.041)
Δ IL-6 0–24 h, %	0.28 (0.102)	−0.24 (0.097)	—	0.06 (0.704)	0.19 (0.199)	0.10 (0.549)
Δ CRP 0–24 h, %	−0.17 (0.322)	−0.03 (0.867)	0.06 (0.704)	—	0.14 (0.337)	0.09 (0.596)
Δ lactate 0–24 h	0.09 (0.601)	−0.13 (0.383)	0.19 (0.199)	0.14 (0.337)	—	−0.05 (0.759)
Δ SOFA 0–48 h	0.36 (0.060)	−0.33 (0.041)	0.10 (0.549)	0.09 (0.596)	−0.05 (0.759)	—

Cells show Spearman’s ρ (*p*-value). Δ VDI is restricted to vasopressor-dependent patients (pairs include 28–35 patients); other pairs include 38–47 patients depending on data availability. Changes in IL-6 and CRP are relative (%); other changes are absolute. Abbreviations and symbols: Δ, change; ρ, Spearman rank correlation coefficient; CRP, C-reactive protein; IL-6, interleukin-6; MAP, mean arterial pressure; SOFA, Sequential Organ Failure Assessment; VDI, vasopressor dependency index.

**Table 7:** Exploratory cross-lagged panel model of lagged associations between interleukin-6, vasopressor dependency index, SOFA score, and lactate across consecutive 0–12, 12–24, and 24–48 h transitions.

Outcome at t + 1	Predictor at t	Standardised β (95% CI)	*p*
IL-6 (log)	IL-6 (log) (autoregressive)	0.66 (0.51 to 0.82)	<0.001
	VDI (log)	−0.05 (−0.20 to 0.09)	0.476
	SOFA	0.18 (0.05 to 0.31)	0.007
	Lactate	0.07 (−0.04 to 0.18)	0.232
VDI (log)	IL-6 (log)	0.04 (−0.04 to 0.13)	0.310
	VDI (log) (autoregressive)	0.69 (0.52 to 0.85)	<0.001
	SOFA	0.19 (0.05 to 0.33)	0.009
	Lactate	0.08 (−0.02 to 0.17)	0.130
SOFA	IL-6 (log)	−0.04 (−0.14 to 0.05)	0.362
	VDI (log)	0.08 (−0.01 to 0.17)	0.095
	SOFA (autoregressive)	0.78 (0.60 to 0.96)	<0.001
	Lactate	0.11 (0.02 to 0.19)	0.015
Lactate	IL-6 (log)	−0.11 (−0.21 to −0.00)	0.042
	VDI (log)	0.00 (−0.17 to 0.18)	0.967
	SOFA	0.07 (−0.05 to 0.19)	0.237
	Lactate (autoregressive)	0.77 (0.66 to 0.89)	<0.001

Standardised regression coefficients (β) with 95% CIs from pooled cross-lagged regressions of each variable at time t + 1 on all four variables at time t and indicators for the transition interval; standard errors clustered by patient (47 patients, 129 transitions). IL-6 and VDI were log-transformed before standardisation. Abbreviations and symbols: β, standardised regression coefficient; t, current time point; t + 1, subsequent time point; CI, confidence interval; IL-6, interleukin-6; SOFA, Sequential Organ Failure Assessment; VDI, vasopressor dependency index. The model describes lagged associations in pooled 12 h and 24 h intervals and does not establish temporal precedence.

## Data Availability

The anonymised dataset analysed in this study is available from the corresponding author upon reasonable request.

## Supplementary Materials

The following supporting information can be downloaded at: https://www.mdpi.com/article/10.3390/biomedicines14102230/s1, Table S1: Subgroup analysis of the association between adsorptive therapy (oXiris or CytoSorb) and the absolute 24 h change in the vasopressor dependency index among vasopressor-dependent patients; Table S2: Desirability-of-outcome ranking (DOOR) at day 28 and pairwise win statistics according to treatment strategy; Table S3: Firth-penalised logistic regression models for 28-day survival and their internally validated performance.

## Abbreviations

Abbreviations are expanded in the second column; II denotes the second version of the APACHE score. The following abbreviations are used in this manuscript:

APACHE II	Acute Physiology and Chronic Health Evaluation II
ARDS	acute respiratory distress syndrome
AUC	area under the receiver operating characteristic curve
BMI	body mass index
CI	confidence interval
CRP	C-reactive protein
CRRT	continuous renal replacement therapy
CVVHDF	continuous veno-venous haemodiafiltration
DOOR	desirability of outcome ranking
ECMO	extracorporeal membrane oxygenation
IL-6	interleukin-6
IQR	interquartile range
KDIGO	Kidney Disease: Improving Global Outcomes
LRT	likelihood-ratio test
MAP	mean arterial pressure
NEE	norepinephrine-equivalent dose
OR	odds ratio
PAES	polyarylethersulfone
PCT	procalcitonin
PMMA	polymethylmethacrylate
SD	standard deviation
SOFA	Sequential Organ Failure Assessment
STROBE	Strengthening the Reporting of Observational Studies in Epidemiology
TRIPOD	Transparent Reporting of a multivariable prediction model for Individual Prognosis Or Diagnosis
VDI	vasopressor dependency index
VIS	vasoactive-inotropic score
