# Supplementary material for: Cytokine and Vasopressor Trajectories During Continuous Renal Replacement Therapy with oXiris, CytoSorb, or Standard Haemofilters in Sepsis-Associated Acute Kidney Injury: An Exploratory Retrospective Cohort Study
Source: Biomedicines. 2026 Oct 2;14(10):2230. doi: 10.3390/biomedicines14102230 (PMC13701235; doi:10.3390/biomedicines14102230)
Supplement: Supplementary file 1 [file biomedicines-14-02230-s001.zip › biomedicines-4597900-supplementary.pdf]

Supplementary Materials

Cytokine and Vasopressor Trajectories During Continuous Renal Replacement Therapy with oXiris, CytoSorb or Standard Haemofilters in Sepsis-Associated Acute Kidney Injury: An Exploratory Retrospective Cohort Study

Tamara Mirela Porosnicu, Ciprian Gindac, Marius Papurica, Alexandru Silvius Pescariu, Felix Bratosin, Elena Hogea, Dorel Sandesc and Ovidiu Bedreag

Table S1. Subgroup analysis of the association between adsorptive therapy (oXiris or CytoSorb) and the absolute 24 h change in the vasopressor dependency index among vasopressor-dependent patients.

Subgroup	n (adsorptive / conventional)	Hodges–Lehmann difference (95% CI)	p	p interaction
All vasopressor-dependent patients	20 / 15	−0.14 (−0.44 to 0.02)	0.077	—
Baseline IL-6: ≥ median	11 / 7	−0.26 (−0.70 to 0.32)	0.425	0.881
Baseline IL-6: < median	9 / 8	−0.08 (−0.46 to 0.07)	0.188	
Baseline SOFA: ≥ 12	14 / 13	−0.14 (−0.53 to 0.07)	0.093	0.831
Baseline SOFA: < 12	6 / 2	−0.11 (−0.46 to 0.34)	0.286	
Age: ≥ 65 years	8 / 9	−0.21 (−0.52 to 0.06)	0.092	0.518
Age: < 65 years	12 / 6	−0.09 (−0.84 to 0.27)	0.451	
Baseline norepinephrine: ≥ 0.3 µg/kg/min	11 / 8	−0.41 (−1.09 to 0.12)	0.148	0.264
Baseline norepinephrine: < 0.3 µg/kg/min	9 / 7	−0.02 (−0.18 to 0.18)	0.631	
Baseline lactate: ≥ 2 mmol/L	12 / 9	−0.36 (−0.84 to 0.03)	0.094	0.405
Baseline lactate: < 2 mmol/L	8 / 6	−0.07 (−0.18 to 0.27)	0.476	

Negative values indicate a larger reduction in VDI with adsorptive therapy (oXiris or CytoSorb) than with conventional CRRT. Hodges–Lehmann median differences with distribution-free 95% CIs; p-values from the Mann–Whitney U test; interaction p-values from linear models on rank-transformed outcomes with a subgroup × treatment term. The IL-6 median among vasopressor-dependent patients was 114 pg/mL. All subgroup analyses are exploratory, unadjusted for multiplicity and underpowered; no stratum should be interpreted as identifying patients who benefit from adsorptive therapy. Abbreviations: CI, confidence interval; CRRT, continuous renal replacement therapy; IL-6, interleukin-6; SOFA, Sequential Organ Failure Assessment; VDI, vasopressor dependency index.

Table S2. Desirability-of-outcome ranking (DOOR) at day 28 and pairwise win statistics according to treatment strategy.

DOOR rank / statistic	Conventional CRRT (n = 22)	oXiris (n = 21)	CytoSorb (n = 5)	Adsorptive, pooled (n = 26)
1. Alive, vasopressor-free at 48 h, SOFA improved ≥ 2	1 (4.5)	3 (14.3)	1 (20.0)	4 (15.4)
2. Alive, vasopressor-free at 48 h, SOFA not improved	0 (0.0)	1 (4.8)	0 (0.0)	1 (3.8)
3. Alive, vasopressor-dependent or unknown status at 48 h	2 (9.1)	3 (14.3)	1 (20.0)	4 (15.4)

4. Died by day 28	19 (86.4)	14 (66.7)	3 (60.0)	17 (65.4)
Mean DOOR rank (lower = better)	3.77	3.33	3.20	3.31
Win probability vs conventional (95% CI)	—	0.603 (0.476–0.716)	0.636 (0.409–0.868)	0.609 (0.492–0.725)
Win ratio vs conventional (95% CI)	—	3.07 (0.69–13.31)	4.00 (0.00–33.00)	3.23 (0.90–13.62)
p vs conventional	—	0.122	0.181	0.091

Values are n (%) unless stated. Ranks are ordered from most (1) to least (4) desirable; patients alive but without 48-h data were assigned rank 3. Win probability = (wins + 0.5 × ties)/all patient pairs; win ratio = wins/losses; 95% CIs from 2000 bootstrap resamples; p-values from the Mann–Whitney U test. Because the conventional comparator was heterogeneous (including five PMMA-treated patients), the CytoSorb group contained five patients and allocation was not random, these statistics are exploratory descriptive summaries and not efficacy estimates. Abbreviations: CI, confidence interval; CRRT, continuous renal replacement therapy; DOOR, desirability of outcome ranking; PMMA, polymethylmethacrylate; SOFA, Sequential Organ Failure Assessment.

Table S3. Firth-penalised logistic regression models for 28-day survival and their internally validated performance.

Model / predictor	OR (95% CI)	p	Apparent AUC	Optimism-corrected AUC	Calibration slope	Brier score (apparent / corrected)
M1: baseline clinical			0.888	0.882	1.09	0.110 / 0.125
Baseline SOFA (per point)	0.59 (0.42 to 0.83)	<0.001				
Age (per 10 years)	0.51 (0.27 to 0.96)	0.020				
M2: M1 + favourable haemodynamic status at 24 h			0.893	0.866	0.92	0.106 / 0.134
Baseline SOFA (per point)	0.66 (0.46 to 0.94)	0.007				
Age (per 10 years)	0.50 (0.26 to 0.96)	0.019				
Favourable haemodynamic status at 24 h	3.21 (0.51 to 20.32)	0.199				
M3: M2 + 24-h IL-6 log-ratio + adsorptive device			0.898	0.842	0.82	0.102 / 0.144
Baseline SOFA (per point)	0.68 (0.48 to 0.97)	0.013				
Age (per 10 years)	0.51 (0.26 to 0.99)	0.027				
Favourable haemodynamic status at 24 h	3.01 (0.45 to 20.05)	0.243				
IL-6 log-ratio 24 h/0 h (per unit)	0.86 (0.48 to 1.56)	0.633				
Adsorptive	2.78 (0.46 to	0.242				

device	16.97)					

Models fitted in 47 patients with 12 survivors. ORs with Wald 95% CIs; p-values from penalised likelihood-ratio tests. Optimism-corrected AUC and Brier score from 500 bootstrap resamples with model refitting; the calibration slope is the mean slope of the linear predictor from bootstrap models applied to the original data. Age is modelled per 10 years; the IL-6 log-ratio is ln(IL-6 at 24 h/IL-6 at 0 h). Models M2 and M3 contain variables measured at 24 h and are therefore 24-h landmark models conditional on survival with available data at 24 h; they are not baseline prediction models. M3 (five predictors for 12 events) falls below the recommended events-per-variable ratio and is shown only to illustrate the increase in apparent performance without improvement in optimism-corrected performance. Abbreviations: M1, baseline SOFA-and-age model; M2, M1 plus favourable haemodynamic status; M3, M2 plus IL-6 log-ratio and adsorptive device; AUC, area under the receiver operating characteristic curve; CI, confidence interval; IL-6, interleukin-6; OR, odds ratio; SOFA, Sequential Organ Failure Assessment.
